# Severe pneumonia and pathogenic damage in human airway epithelium caused by Coxsackievirus B4

**DOI:** 10.1080/22221751.2023.2261560

**Published:** 2023-09-27

**Authors:** Jing Dai, Duo Xu, Chao Yang, Huan Wang, Dehui Chen, Zhengshi Lin, Shuyan Qiu, Li Zhang, Xiao Li, Xingui Tian, Qian Liu, Yujun Cui, Rong Zhou, Wenkuan Liu

**Affiliations:** a State Key Laboratory of Respiratory Diseases, National Clinical Research Center for Respiratory Disease, Guangdong-Hong Kong-Macao Joint Laboratory of Respiratory Infectious Disease, The First Affiliated Hospital of Guangzhou Medical University, Guangzhou Institute of Respiratory Health, Guangzhou Medical University, Guangzhou, People’s Republic of China; b The Center for Microbes, Development and Health, CAS Key Laboratory of Molecular Virology and Immunology, Institut Pasteur of Shanghai, Chinese Academy of Sciences, Shanghai, People’s Republic of China; c State Key Laboratory of Pathogen and Biosecurity, Beijing Institute of Microbiology and Epidemiology, Beijing, People’s Republic of China; d Scientific Research Center, The First Affiliated Hospital of Guangdong Pharmaceutical University, Guangdong Pharmaceutical University, Guangzhou, People’s Republic of China

**Keywords:** Coxsackievirus B4, severe pneumonia, hand-foot-mouth disease (HFMD), pathogenic damage, human airway epithelium, full-length genome, neurovirulence

## Abstract

Coxsackievirus B4 (CVB4) has one of the highest proportions of fatal outcomes of other enterovirus serotypes. However, the pathogenesis of severe respiratory disease caused by CVB4 infection remains unclear. In this study, 3 of 42 (7.2%, GZ-R6, GZ-R7 and GZ-R8) patients with severe pneumonia tested positive for CVB4 infection in southern China. Three full-length genomes of pneumonia-derived CVB4 were sequenced and annotated for the first time, showing their high nucleotide similarity and clustering within genotype V. To analyze the pathogenic damage caused by CVB4 in the lungs, a well-differentiated human airway epithelium (HAE) was established and infected with the pneumonia-derived CVB4 isolate GZ-R6. The outcome was compared with that of a severe hand-foot-mouth disease (HFMD)-derived CVB4 strain GZ-HFM01. Compared with HFMD-derived CVB4, pneumonia-derived CVB4 caused more intense and rapid disruption of HAE polarity, leading to tight-junction barrier disruption, loss of cilia, and airway epithelial cell hypertrophy. More pneumonia-derived CVB4 were released from the basolateral side of the HAE than HFMD-derived CVB4. Of the 18 cytokines tested, only IL-6 and IL-1b secretion significantly increased on bilateral sides of HAE during the early stage of pneumonia-derived CVB4 infection, while multiple cytokine secretions significantly increased in HFMD-derived CVB4-infected HAE. HFMD-derived CVB4 exhibited stronger neurovirulence in the human neuroblastoma cells SH-SY5Y than pneumonia-derived CVB4, which is consistent with the clinical manifestations of patients infected with these two viruses. This study has increased the depth of our knowledge of severe pneumonia infection caused by CVB4 and will benefit its prevention and treatment.

## Introduction

Coxsackieviruses (CV), which belong to the genus *Enterovirus* within the family *Picornaviridae*, are small non-enveloped RNA viruses with a single-stranded positive-sense genome of approximately 7400 nucleotides (nt) [1]. They are associated with various diseases, such as paralytic diseases, hand-foot-mouth disease (HFMD), meningitis-encephalitis, myocarditis, febrile diseases, and respiratory infections [2]. According to their pathogenicity in suckling mice and genome diversity, CV can be divided into two groups: A (CVA) and B (CVB) [3]. To date, evidence shows CVA has 21 serotypes, and many CVA types, like CVA16, CVA6, and CVA10, can cause HFMD [4–7]. CVB has 6 types (CVB1–6), which are associated with a variety of severe diseases, including severe central nervous system and heart-related diseases [8,9].

CVB4 is an important type of CVB with global distribution and endemic circulation [10]. It is associated with one of the highest number of reports of fatal outcomes, with a significantly higher risk of death compared to all other enterovirus (EV) serotypes [11]. CVB4 has been reported to be closely associated with aseptic meningitis, myocarditis, HFMD, acute pancreatitis, and type I diabetes [12–15]. However, the severe respiratory diseases caused by CVB4 infection and its pathogenesis are rarely reported [16–18]. There is still no vaccine for CVB4. Although positive progress has been made in research into CVB4 infectious disease drugs, such as fluoxetine [19,20] and bacterial secreted protein [21,22], there are no specific drugs available for clinical use at present, and symptomatic treatment is the main treatment method.

CVB4 comprises five genotypes, genotypes I – V, according to the type of VP1 gene [10]. Research has shown that CVB4 might have undergone intertypic recombination with other EV-B strains [13,23], and genotype V has high variability [24]. Previous transmission dynamics analysis based on the VP1 region revealed that CVB4 in European and American countries is dominated by genotype IV, while genotype V is absolutely dominant in China [13,14,24]. However, in 2019, an imported recombinant virus belonging to genotype IV was isolated from a patient with HFMD in Tianjin [25]. Given the high lethality and variability of CVB4, the monitoring of CVB4 infections needs to be taken seriously.

In this study, we analyzed CVB4 infections in patients with severe pneumonia in Guangzhou, China. Positive samples were isolated using susceptible cells screened from multiple types of permissive cells, and the full-length genome sequences of pneumonia-derived CVB4 isolates were obtained for the first time in this work using next-generation sequencing. The pathogenic characteristics of the CVB4 isolates were explored using tissue-like human airway epithelial (HAE) cells. This study provides important knowledge for an in-depth understanding of CVB4 and provides a reference for research into its treatment, prevention, and control.

## Materials and methods

### Ethics statement

The study was approved by the First Affiliated Hospital of Guangzhou Medical University Ethics Committee. Next of kin, caretakers, or guardians provided written informed consent for participation in the study on behalf of all minors/children.

### Patients with severe pneumonia and sample collection

A total of 42 paediatric patients (≤ 14 years old) with severe pneumonia, identified by attending physicians as previously reported [26–28], were enrolled, and respiratory samples (e.g. throat swabs, sputum, and bronchoalveolar lavage fluid) were collected in accordance with established clinical protocols at the First Affiliated Hospital of Guangzhou Medical University between April and September 2018 in Guangzhou, southern China [29]. The samples were refrigerated at 2–8 °C in viral transport medium, transported on ice to the State Key Laboratory of Respiratory Diseases, and analyzed immediately or stored at −80 °C before analysis.

### CVB4 and common respiratory pathogen screening

EV screening was conducted using pan-EV TaqMan real-time PCR (qPCR) and EV typing was performed using EVA71, CVA16 and CVB4 qPCR methods, as well as the sequencing analysis of VP1 fragment (450 bp) amplified with typing primers. The specific primers and probe were shown in Supplementary Table S1. The qPCR protocol was conducted using our optimized reaction buffer and cycling conditions as explained in a previous report [30]. The VP1 fragment amplification was performed using a one-step RT–PCR kit (RR055A, TaKaRa, Dalian, China) according to the supplier’s instructions. The RT–PCR protocol was as follows: 50°C for 30 min and 94°C for 2 min, followed by 35 cycles of 94°C for 40 s, 52°C for 40 s and 72°C for 40 s. CVB4-positive samples were subjected to testing for 17 other common respiratory pathogens, including influenza A virus, influenza B virus, respiratory syncytial virus, parainfluenza virus types 1–4, human metapneumovirus, human rhinovirus, four types of coronaviruses (229E, OC43, NL63, and HKU1), human adenovirus, human bocavirus, *Mycoplasma pneumoniae*, and *Chlamydophila pneumoniae,* as previously reported [29].

### Clinical presentation collection

The clinical characteristics, treatments, and outcomes of patients with CVB4 infections were retrospectively collected from their medical records.

### Culture of pneumonia-derived CVB4 isolates with susceptible cells

Ten kinds of cells, human rhabdomyosarcoma (RD), LLC-MK2, HEp-2, Hela, MDCK, BHK-21, A549, HEK293, Vero-E6, and MRC-5 (ATCC, USA, stored in the State Key Laboratory of Respiratory Diseases), were used for CVB4-positive isolate culture. LLC-MK2 and HEp-2 were cultured in RPMI 1640 medium (Gibco, Beijing, China) supplemented with 10% (v/v) fetal bovine serum (FBS; ExCell Bio, Taicang, China) and 1% penicillin–streptomycin (P/S) (Gibco, NY, USA); RD, Hela, MDCK, BHK-21, A549, HEK293 and Vero-E6 were cultured in Dulbecco’s modified essential medium (DMEM; Gibco, Beijing, China) + 10% FBS + 1% P/S; MRC-5 was cultured in minimum essential medium (MEM; Gibco, Beijing, China) + 10% FBS + 1% P/S. The cells were grown at 37 °C in an atmosphere of 5% (v/v) CO_2_. CVB4-positive samples were cultured in the cells at 37 °C and 5% CO_2_ and were then maintained in the respective basal medium supplemented with 2% FBS + 1% P/S. Inoculated cells were monitored daily for cytopathic effect (CPE); they were harvested at almost full CPE. If there was no CPE, blind passage was continued to the third generation.

### Severe HFMD-derived CVB4 stock

The CVB4 epidemic strain GZ-HFM01, which was isolated from stool samples of a two-year-old male patient with severe HFMD (with aseptic meningitis) in 2015, was generously provided by Professor Xun Zhu of Sun Yat-sen University and cultured using recommended LLC-MK2 cells. The HFMD-derived CVB4 strain GZ-HFM01 and pneumonia-derived CVB4 isolates in this study were cultured for analysis of genome features and infectious and pathogenic properties.

### CVB4 genome sequencing and annotation

CVB4-positive isolates and GZ-HFM01 were cultured and harvested for genomic sequencing. Viral RNA was extracted using a TaKaRa Mini BEST Viral RNA/DNA Extraction Kit Ver. 5.0 (TaKaRa, Dalian, China), in accordance with the manufacturer’s instructions. Genomes were analyzed by next-generation sequencing using an Illumina NovaSeq 6000 sequencer in accordance with a protocol from Synbio-Technologies (paired-end, 2 × 150 bp). The complete genomes of CVB4 isolates were assembled using CLC Genomics Workbench 11.0 (Qiagen, Germantown, MD, USA). The complete genomes of the CVB4 isolates and GZ-HFM01 were annotated and uploaded to the GenBank database.

### Phylogenetic analysis

All genomic sequences of CVB4 with complete ORFs were deposited in the GenBank database (as of May 1, 2023), and typical strains of CVB4 genotypes I – V sequences of VP1 genes were retrieved from GenBank for phylogenetic analyses with taxon names that included the genome type, corresponding GenBank accession number, country of isolation, strain name, and year of isolation. Phylogenetic analysis was performed using Molecular Evolutionary Genetics Analysis (MEGA) version 11.0.8. Phylogenetic trees were constructed by the neighbour-joining method with 1,000 bootstrap replicates and default settings for all other parameters [31].

### Viral plaque formation assay

Hela and human neuroblastoma cells SH-SY5Y (stored in the State Key Laboratory of Respiratory Diseases) were cultured for analysis of the cytopathogenesis properties of pneumonia-derived CVB4 isolates, and the findings were compared with those for HFMD-derived CVB4 strain GZ-HFM01. Cells were seeded into six-well culture plates and incubated overnight to form dense monolayers with more than 90% confluence. After removal of the growth media, the cultures were inoculated with 0.4 mL of 10-fold serial dilutions of the viral stocks and were then incubated for 1 h at 37 °C with rocking every 15 min. The viral inoculant was removed by aspiration, and 3 mL DMEM-agarose mulch [2% SeaPlaque GTG-agarose (Lonza, Rockland, USA) mixed 1:1 with 2 × DMEM medium containing 4% FBS] was added to each well. The agarose was allowed to solidify at room temperature (20–26 °C). Plaque plates were incubated at 37 °C and 5% CO_2_ for 2 days (for Hela) and 3 days (for SH-SY5Y). Plates were stained with 2 mL/well 20% ethanol, 2% paraformaldehyde, and 1% crystal violet overnight at room temperature. The diameters of the plaques were measured using the VisionWorks software package (Analytik Jena, Jena, Germany).

### Human airway epithelium culture

Normal human primary bronchial/tracheal epithelial cells (NHBE) cells derived from an 8-year-old female who tested negative for bacteria, yeast, fungi, *mycoplasma*, hepatitis B, hepatitis C and HIV were purchased from Lifeline (Lifeline, Frederick, MD, USA). Un-differentiated NHBE cells were cultured and passaged according to instructions provided by the supplier with serum-free medium (BronchiaLife B/T complete medium, Lifeline, USA). The polarity of HAE cells was differentiated from that of NHBE cells at the air–liquid interface (ALI) on collagen-coated Transwell inserts (0.3 cm^2^, 0.4 μm pore size, BD-Falcon, Tewksbury, MA, USA) in 24-well plates at 37 °C, 5% CO_2_ in differentiation medium for 21 days, and determined by transepithelial electrical resistance (TEER) measurement using two Millicell-ERS (MERS00002, Millipore, Burlington, MA, USA) electrodes submerged in the media in the apical and basolateral chambers of the inserts, according to our previous report [32]. HAE cells with TEER values >1000 Ω^.^cm^2^ were considered well-differentiated and met the requirement for subsequent studies with the model [33–35].

### CVB4 infection in HAE

Well-differentiated HAE were cultured on inserts in 24-well plates (0.3 cm^2^) and inoculated with the typical CVB4 isolate GZ-R6 at a multiplicity of infection (MOI) of 0.5 from the apical surface. The HFMD-derived strain GZ-HMF01 and PBS (mock infection) were used for comparative analyses. For each HAE, 2 h viral incubation was followed by aspiration of the viruses from the apical chamber and by three washes of the cells with 200 μL of PBS to remove unbound virus. The HAEs were then further cultured at an ALI.

### Proliferation curve of CVB4

The proliferation dynamics of a typical pneumonia-derived CVB4 isolate in SH-SY5Y was analyzed, and CVB4 qPCR was used to quantify the CVB4 strains. HFMD-derived strain GZ-HFM01 was analyzed simultaneously. SH-SY5Y cells (80% confluence) were infected with CVB4 stains at an MOI of 0.01 in 96-well culture plates at 37 °C with 5% CO_2_. After virus adsorption for 2 h, the virus supernatant was aspirated, and cells were washed twice with DMEM to remove unabsorbed virus. Maintenance solution (0.2 mL/well; DMEM + 2% FBS) was added for subsequent culture. Infected cells were then harvested at 24, 48, 72, 96, 120, 144 and 168 h post infection (h.p.i.). Viral genome copies were quantified using CVB4 qPCR.

HAE were infected CVB4 at an MOI of 0.5 in 24-well Transwell plates. Apical washing samples and basolateral medium samples were collected daily from both the apical and basolateral surfaces of HAE until 12 days post-infection (d.p.i.). Apical washing and harvesting were performed by adding 200 μL of differentiation medium to the apical chamber, incubating the samples for 10 min at 37 °C and 5% CO_2_, and removing and storing the 200 μL of differentiation medium from the apical chamber. A 200 μL aliquot of differentiation medium of basolateral chamber was also harvested for virus tests, and another 200 μL of fresh differentiation medium were re-added. A 50 μL aliquot of each virus sample was used for viral RNA extraction and quantification using CVB4 qPCR.

### Cytokine testing

To analyze the immune changes caused by CVB4 infection, a Human Magnetic Luminex Assay (18 Plex, Luminex, R&D Systems, ThermoFisher, USA) was conducted to detect important inflammatory markers or chemokines related to infection in both apical washing samples and basolateral medium samples at 2, 4, 6, 8, 10 and 12 d.p.i., including IL-1b (IL-1β), IL-2, IL-4, IL-6, IL-8, IL-10, IL-27, IL-33, IL-18, TNF-α, IFN-γ, IFN-α, TIMP-1, CCL5 (RANTES), CCL2 (MCP-1), CXCL6, CD54 (ICAM-1), and CD106 (VCAM-1), and sixth day samples from the mock infection culture were used for negative controls. The testing was conducted according to the supplier's instructions using Luminex 200 with xPONENT (Luminex, R&D Systems, USA).

### Immunofluorescence assay

Two biomarkers of HAE – the tight junction protein zona occludens-1 (ZO-1) [33] and cilia marker α-tubulin [36] – were tested for damage to HAE infected with CVB4 at 4 and 8 d.p.i. by immunofluorescence assay (IFA). HAE insert membranes were fixed with cold absolute ethanol for 20 min. Fixed membranes were cut into several small pieces, washed in PBS three times for 5 min, and permeabilized with 0.2% Triton X-100 for 15 min at room temperature. Membranes were blocked with 10% goat serum for 30 min at room temperature, then incubated overnight at 4°C with primary CVB4 – (#MAB941, Merck, USA), ZO-1 – (#13663, CST, Danvers, MA, USA), and α-tubulin-specific (#2125, CST, USA) antibodies diluted 1:200 to 1:1000 in PBS plus 2% goat serum, according to the suppliers recommendations. Subsequently, membranes were incubated with a fluorescein Alexa fluor 488-conjugated secondary antibody (#4412, CST) or Alexa Fluor 594-conjugated secondary antibody (#ab150116, Abcam). The cell nuclei were stained with DAPI. Confocal images were captured using a Zeiss LSM880 with Airyscan confocal microscope (Zeiss, Germany) controlled by ZEISS ZEN 3.4 software.

### Histology analysis

HAE insert membranes infected with CVB4 at 8 d.p.i. were washed with PBS and fixed in 4% paraformaldehyde for 30 min. The fixed membranes were cut into several small pieces and washed with PBS three times. Each membrane fragment was transferred to 20% sucrose in a 15-ml conical tube and allowed to drop to the bottom; it was then embedded vertically in paraffin in an orientation that enabled sectioning of the membrane perpendicular to the blade. The sections were cut at a thickness of 8 μm, placed onto slides, and stained with hematoxylin and eosin (H&E). Images were taken with Precice 500B (Medite, Germany).

### Statistical analysis

Statistical analysis was performed using GraphPad Prism version 7 (GraphPad, San Diego, CA, USA). Differences between groups were calculated using the ANOVA and Mann – Whitney *U* test. A *p*-value of < 0.05 was considered statistically significant.

## Results

### Clinical characteristics of patients with severe pneumonia and CVB4 infection

Three of the 42 severe pneumonia patients (7.1%) (GZ-R6, GZ-R7, and GZ-R8), aged 6, 0.33, and 0.85 years old, respectively, tested positive for EV infection and their type was confirmed as CVB4. There were no incidences of the 17 other common respiratory viruses of concern in this study, and no bacterial or fungal infections were found according to the clinical records. The main clinical manifestation was severe pneumonia, and chest X-rays showed pneumonia. All three patients’ indexes of IL-6, platelets, procalcitonin, aspartate aminotransferase and lactate dehydrogenase were higher than the normal ranges. There were no signs of lethargy or fatigue, no rash on the oral mucosa, hands, feet, or buttocks, and no other underlying diseases. The duration of the disease was relatively long (26 to 129 days) (Table 1).

**Table 1.** Clinical characteristics, treatments, and outcomes of the three patients infected with coxsackievirus B4.

Characteristic	Coxsackievirus B4-positive patient		
	GZ-R6	GZ-R7	GZ-R8
**Diagnosis of physician**	Severe pneumonia	Severe pneumonia	Severe pneumonia
**Radiologic findings**	Pneumonia	Pneumonia	Pneumonia
**Clinical characteristic**			
Sex	Female	Male	Male
Age, year	6	0.33	1.58
Existing chronic disease	Negative	Negative	Negative
Initial symptom	Dyspnea + cough	Cough	Dyspnea + cough
The highest temperature, ^o^C	37.7	38.7	40
Shortness of breath	Positive	Positive	Positive
Rash	Negative	Negative	Negative
Fatigue	Negative	Negative	Negative
Headache	Negative	Negative	Negative
**Laboratory findings**			
The blood oxygen saturation under inhalation, %	95	94	95
Bacteria or fungus culture	Negative	Negative	Negative
White-cell count, × 10^9^/L	12 ↑	12.92 ↑	9.51
Lymphocyte count, × 10^9^/L	3	2.2	0.3 ↓
Platelet count, × 10^9^/L	**679 ↑**	**794 ↑**	**402 ↑**
Hemoglobin, g/L	142	84 ↓	89 ↓
Procalcitonin, ng/mL	**0.1 ↑**	**0.11 ↑**	**0.1 ↑**
Alanine aminotransferase, U/L	83.7 ↑	20.6	217.3 ↑
C-reactive protein, mg/dL	1.2 ↑	1.36 ↑	0.01
Aspartate aminotransferase, U/L	**58.1 ↑**	**47 ↑**	**100.5 ↑**
Creatine kinase, U/L	101.6	233 ↑	24.2
Lactate dehydrogenase, U/L	**333.6 ↑**	**277 ↑**	**305.7 ↑**
D-dimer, ng/mL	213	350	320
Interleukin – 6, pg/mL	**29.1 ↑**	**84.75 ↑**	**7.26 ↑**
**Treatments**			
Symptomatic treatment	Anti-infection, anti-inflammation, intravenous fluid therapy, atomization inhalation treatment	Anti-infection, anti-inflammation, intravenous fluid therapy	Anti-infection, anti-inflammation, intravenous fluid therapy
Mechanical ventilation	No	No	Yes
**Clinical outcomes – recovery duration**			
Total disease duration, day	26	129	120
Length of hospital stay, day	16	46	14

Normal index range of test items: white cell count, 4–10 × 10^9^/L; lymphocyte count, 0.9–5.2 × 10^9^/L; platelet count, 100–400 × 10^9^/L; hemoglobin, 120–150 g/L; procalcitonin, 0–0.05 ng/mL; alanine aminotransferase, 5–40 U/L; C-reactive protein, 0–0.6 mg/dL; aspartate aminotransferase, 5–40 U/L; creatine kinase, 10–190 U/L; lactate dehydrogenase, 109–255 U/L; D-dimer, 68–494 ng/mL; Interleukin-6, 0–5.30 pg/mL. “↑”, above the upper limit of the normal range; “↓”, below the lower limit of the normal range. The indexes that showed higher than normal ranges in all three patients are indicated in bold.

### Cells susceptible to pneumonia-derived CVB4 culture

Throat swab samples from 3 CVB4-positive patients (Ct values ranged from 25 to 29) were used for viral culture. However, permissive cells commonly used to culture CVB4 from stool or cerebrospinal fluid, such as LLC-MK2 or RD cells [37], were not successfully cultivated. Therefore, in this study, eight other kinds of cell lines were used to select cells susceptible to pneumonia-derived CVB4 inoculation. After blind passaging for three times, CPE were observed only in Hela cells, and the CPE characteristics were consistent with the HFMD-derived CVB4 strain GZ-HFM01, including typical cytolytic properties (Supplementary Figure S1).

### Full-length genome of CVB4 obtained for the first time from respiratory disease patient

**Figure 1.**
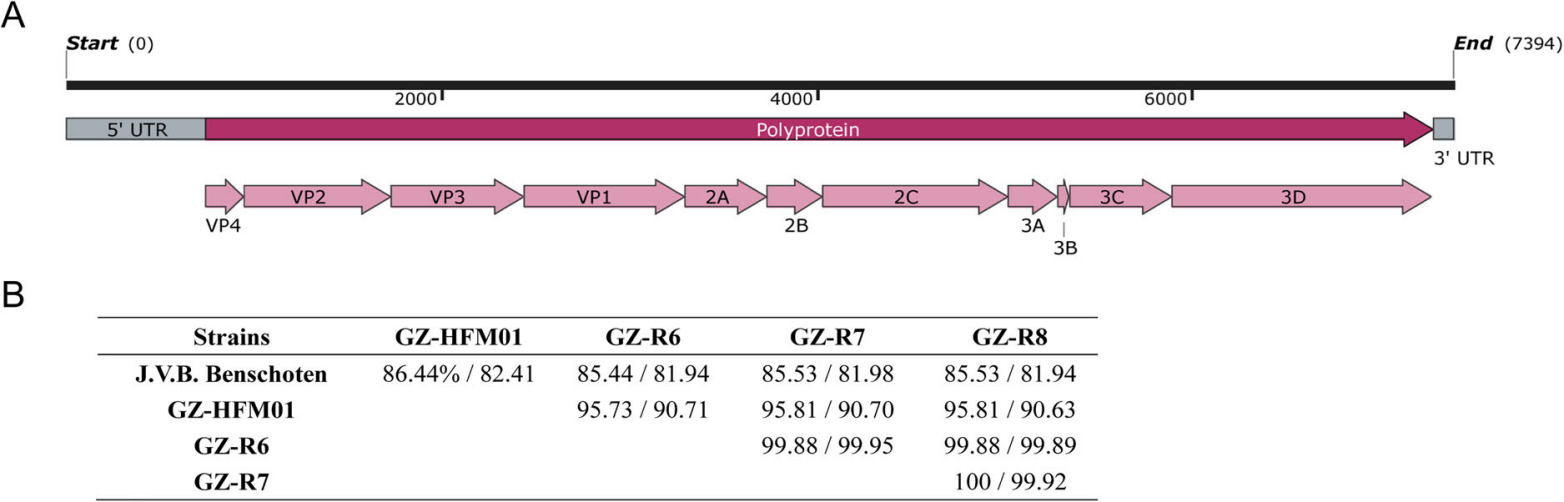
The genome organization (A) and percent identities of VP1/genome sequences (B) among pneumonia-derived/HFMD-derived coxsackievirus B4 isolates and prototype J.V.B. Benschoten. The genome is indicated by the black horizontal line marked at 2000-nt intervals. Untranslated regions (UTR) at both ends are designated by gray boxes; the dark red arrow indicates the coding sequence; while light red arrows designate mature peptides. Percent identities are showed as VP1/genome.

For the three pneumonia-derived and one HFMD-derived CVB4 genome sequencing isolates, contigs and consensus sequences were acquired using CLC Genomics Workbench v11.0 based on CVB4 prototype J.V.B. Benschoten (accession number X05690). About 10 million reads were obtained per sample, resulting in a sequencing depth of 30× to 500× coverage of the entire genome. A total of 7394 nucleotides (nt) of the genomes of the four isolates were obtained in this study, including a 741-nt 5′ untranslated region (5′-UTR), a 101-nt 3′-UTR, and an open reading frame (ORF) (6552 nt). The ORF encodes a polyprotein of 2183 amino acids (aa), which is then digested to form 11 mature peptides (Figure 1A). The genome sequences of GZ-HFM01 and GZ-R6 to – R8 were submitted to Genbank with accession no. MZ540957 – MZ540960, respectively. Based on the nucleotide alignment of VP1 and genomic sequences, three pneumonia-derived CVB4 isolates (GZ-R6 to – R8) obtained in this research had high nucleotide identity >99.88% and shared about 85.44% – 85.53% (VP1) and 82.94% – 82.98% (genome) nucleotide identity with the prototype J.V.B. Benschoten and 95.73% – 95.81% (VP1) and 90.63% – 90.71% (genome) nucleotide identity with HFMD-derived strain GZ-HFM01 genomes (Figure 1B). Compared with the prototype strain J.V.B. Benschoten, which has a full-length genome of 7395 nt, the four CVB4 strains sequenced in this research had 2-nt shorter and 1-nt longer 5′-UTR and 3′-UTR, respectively, and the mutations are labelled in Supplementary Table S2. The corresponding non-synonymous mutation sites in the ORF of J.V.B. Benschoten, GZ-HFM01 and GZ-R6 to – R8 are listed in Table 2. There were 84 aa mutations in the 5 strains of concern and 40 aa mutations between the pneumonia-derived CVB4 isolates and the HFMD-derived strain GZ-HFM01. The virulent determinants Thr-129 VP1 and Arg-16 VP4, which have been proven to be associated with the development of hypoglycemia and hyperamylasemia [38,39], were not identified in the capsid protein, but Met-129 VP1 and Ser-16 VP4 were found in GZ-HFM01 and the three pneumonia-derived CVB4 isolates.

**Table 2.** Comparative nonsynonymous mutation site of pneumonia-derived coxsackievirus B4 isolates GZ-R6, GZ-R7 and GZ-R8 with the reference HFMD-derived strain GZ-HFM01 and the prototype J.V.B. Benschoten.

Genome region	nt (aa)	J.V.B. Benschoten	GZ-HFM01	GZ-R6	GZ-R7	GZ-R8
**VP4**	803/804(20)	AGT(S)	ACC(T)	ACT(T)	ACT(T)	ACT(T)
**VP2**	1419(225)	GAA(E)	**GAC(D)**	**GAG(E)**	**GAG(E)**	**GAG(E)**
	1438/1440(232)	AAG(K)	GAA(E)	GAA(E)	GAA(E)	GAA(E)
	1453/1455(237)	ACG(T)	**GCA(A)**	**ACG(T)**	**ACG(T)**	**ACG(T)**
**VP3**	1897/1899(385)	ATC(I)	CTT(L)	CTT(L)	CTT(L)	CTT(L)
	2434/2435(574)	ACA(T)	GAA(E)	GAA(E)	GAA(E)	GAA(E)
**VP1**	2464/2466(574)	TCC(S)	GCT(A)	GCT(A)	GCT(A)	GCT(A)
	2638(632)	ATA(I)	GTA(V)	GTA(V)	GTA(V)	GTA(V)
	3236(831)	AGT(S)	AAT(N)	AAT(N)	AAT(N)	AAT(N)
	3248(835)	GAT(D)	**GAT(D)**	**GGT(G)**	**GGT(G)**	**GGT(G)**
**2A**	3296/3297(851)	CCC(*P*)	CTT(L)	CTT(L)	CTT(L)	CTT(L)
	3298/3299/3300(852)	TAT(Y)	**CTA(L)**	**CAA(Q)**	**CAA(Q)**	**CAA(Q)**
	3301(853)	GGA(G)	**GGA(G)**	**AGA(R)**	**AGA(R)**	**AGA(R)**
	3306(854)	CAT(H)	CAA(Q)	CAA(Q)	CAA(Q)	CAA(Q)
	3310(856)	TCA(S)	GCA(A)	GCA(A)	GCA(A)	GCA(A)
	3340(866)	GTA(V)	ATA(I)	ATA(I)	ATA(I)	ATA(I)
	3367/3368/3369(875)	GTG(V)	**GCC(A)**	**ACC(T)**	**ACC(T)**	**ACC(T)**
	3379/3380(879)	AAT(N)	**GGT(G)**	**GAT(D)**	**GAT(D)**	**GAT(D)**
	3482/3483(913)	TTT(F)	TAC(Y)	TAC(Y)	TAC(Y)	TAC(Y)
	3494/3495(917)	AAG(K)	AGA(R)	AGG(R)	AGG(R)	AGG(R)
	3497/3498(918)	AGC(S)	AAT(N)	AAT(N)	AAT(N)	AAT(N)
	3595(951)	ACA(T)	GCA(A)	GCA(A)	GCA(A)	GCA(A)
	3650/3651(969)	GTC(V)	**GCG(A)**	**GTC(V)**	**GTC(V)**	**GTC(V)**
	3682/3684(980)	GTG(V)	ATT(I)	ATT(I)	ATT(I)	ATT(I)
	3700/3702(986)	GTC(V)	**ATT(I)**	**GTT(V)**	**GTT(V)**	**GTT(V)**
**2B**	3810(1022)	GAG(E)	**GAT(D)**	**GAA(E)**	**GAA(E)**	**GAA(E)**
	3818/3819(1025)	AAC(N)	**AAT(N)**	**AGT(S)**	**AGT(S)**	**AGT(S)**
	3933(1063)	ATC(I)	**ATG(M)**	**ATA(I)**	**ATA(I)**	**ATA(I)**
	3970/3972(1076)	TCG(S)	**TCA(S)**	**ACT(T)**	**ACT(T)**	**ACT(T)**
	3996(1084)	CAC(H)	**CAG(Q)**	**CAT(H)**	**CAT(H)**	**CAT(H)**
	4003/4005(1087)	TCC(S)	**TCA(S)**	**GCC(A)**	**GCC(A)**	**GCC(A)**
	4013/4014(1090)	TAC(Y)	**TTT(F)**	**TAC(Y)**	**TAC(Y)**	**TAC(Y)**
**2C**	4045/4047(1101)	GGG(G)	**GGG(G)**	**AGC(S)**	**AGC(S)**	**AGC(S)**
	4105/4107(1121)	GTC(V)	**ATC(I)**	**GTG(V)**	**GTG(V)**	**GTG(V)**
	4181/4182(1146)	AGT(S)	**ACC(T)**	**AAC(N)**	**AAC(N)**	**AAC(N)**
	4345/4347(1201)	TCC(S)	**ACC(T)**	**TCT(S)**	**TCT(S)**	**TCT(S)**
	4363/4365(1207)	AGC(S)	**GGT(G)**	**AGC(S)**	**AGC(S)**	**AGC(S)**
	4643/4644(1300)	CCA(*P*)	**CCA(*P*)**	**CCG(*P*)**	**CAG(Q)**	**CCG(*P*)**
	4851(1369)	GAA(E)	**GAG(E)**	**GAT(D)**	**GAT(D)**	**GAT(D)**
	4925/4926(1394)	AAG(K)	AGA(R)	AGA(R)	AGA(R)	AGA(R)
**3A**	5062/5064(1440)	ACA(T)	**ACA(T)**	**GCG(A)**	**GCG(A)**	**GCG(A)**
	5087/5088(1448)	GTA(V)	GCG(A)	GCA(A)	GCA(A)	GCA(A)
	5118(1458)	AGG(R)	AGT(S)	AGT(S)	AGT(S)	AGT(S)
	5119/5121(1459)	CAG(Q)	GAA(E)	GAG(E)	GAG(E)	GAG(E)
	5125/5127(1461)	ATT(I)	GTG(V)	GTC(V)	GTC(V)	GTC(V)
	5147/5148(1468)	AAG(K)	**AGA(R)**	**AAG(K)**	**AAG(K)**	**AAG(K)**
	5167(1475)	ATC(I)	**GTC(V)**	**ATC(I)**	**ATC(I)**	**ATC(I)**
	5170/5172(1476)	GAT(D)	AAT(N)	AAC(N)	AAC(N)	AAC(N)
	5177/5178(1478)	ATT(I)	ACT(T)	ACA(T)	ACA(T)	ACA(T)
**3B**	5329/5331(1529)	GTG(V)	**ATA(I)**	**GTG(V)**	**GTG(V)**	**GTG(V)**
**3C(Vpg)**	5365/5367(1541)	GCT(A)	**TCC(S)**	**GCA(A)**	**GCA(A)**	**GCA(A)**
	5401(1553)	TCC(S)	GCC(A)	GCC(A)	GCC(A)	GCC(A)
	5411/5412(1556)	GTG(V)	**GTA(V)**	**GCG(A)**	**GCG(A)**	**GCG(A)**
	5527/5529(1595)	CTG(L)	GTT(V)	GTA(V)	GTA(V)	GTA(V)
	5545/5547(1601)	ATA(I)	GTG(V)	GTG(V)	GTG(V)	GTG(V)
	5552/5553(1603)	AGA(R)	AAG(K)	AAA(K)	AAA(K)	AAA(K)
	5590/5592(1616)	AAC(N)	**AAT(N)**	**GAC(D)**	**GAC(D)**	**GAC(D)**
	5632/5633/5634(1630)	AAG(K)	CGT(R)	AGG(R)	AGG(R)	AGG(R)
	5642/5643(1633)	GTG(V)	**GCT(A)**	**GTA(V)**	**GTA(V)**	**GTA(V)**
	5659/5661(1639)	GTC(V)	**ATA(I)**	**GTA(V)**	**GTA(V)**	**GTA(V)**
	5710/5711/5712(1656)	AGG(R)	CAA(Q)	CAA(Q)	CAA(Q)	CAA(Q)
	5876(1711)	GGC(G)	GCC(A)	GCC(A)	GCC(A)	GCC(A)
	5884/5885/5886(1714)	AAG(K)	CGT(R)	CGA(R)	CGA(R)	CGA(R)
**3D**	5940(1732)	GAT(D)	GAA(E)	GAA(E)	GAA(E)	GAA(E)
	5972(1743)	AGA(R)	AAA(K)	AAA(K)	AAA(K)	AAA(K)
	6004(1754)	GTC(V)	**ATC(I)**	**GTC(V)**	**GTC(V)**	**GTC(V)**
	6062/6063(1773)	GTC(V)	GCT(A)	GCC(A)	GCC(A)	GCC(A)
	6064(1774)	AAC(N)	**GAC(D)**	**AAC(N)**	**AAC(N)**	**AAC(N)**
	6086/6087(1781)	TTC(F)	TCA(S)	TCA(S)	TCA(S)	TCA(S)
	6131/6132(1796)	CTA(L)	CAG(Q)	CAG(Q)	CAG(Q)	CAG(Q)
	6179(1812)	AAC(N)	AGC(S)	AGC(S)	AGC(S)	AGC(S)
	6186(1814)	GAG(E)	**GAT(D)**	**GAA(E)**	**GAA(E)**	**GAA(E)**
	6347(1868)	AAG(K)	**AGG(R)**	**AAG(K)**	**AAG(K)**	**AAG(K)**
	6496(1918)	GCA(A)	ACA(T)	ACA(T)	ACA(T)	ACA(T)
	6553/6555(1937)	GTT(V)	CTA(L)	CTA(L)	CTA(L)	CTA(L)
	6668/6669(1975)	GAA(E)	**GAG(E)**	**GGG(G)**	**GGG(G)**	**GGG(G)**
	6682(1980)	ACA(T)	TCA(S)	TCA(S)	TCA(S)	TCA(S)
	6842(2033)	AAG(K)	**AGG(R)**	**AAG(K)**	**AAG(K)**	**AAG(K)**
	6916/6917/6918(2058)	TGG(W)	CAC(H)	CAC(H)	CAC(H)	CAC(H)
	6956/6957(2071)	GAC(D)	GGG(G)	GGG(G)	GGG(G)	GGG(G)
	7111/7113(2123)	ATC(I)	**ATC(I)**	**GTT(V)**	**GTT(V)**	**GTT(V)**
	7191(2149)	CAC(H)	CAA(Q)	CAG(Q)	CAG(Q)	CAG(Q)
	7210/7211/7212(2156)	CAA(Q)	TCA(S)	TCC(S)	TCC(S)	TCC(S)
	7249/7251(2169)	CTG(L)	**TTA(L)**	**ATA(I)**	**ATA(I)**	**ATA(I)**

Note: Differences in nucleic acid and amino acid sequences in nonsynonymous mutation sites are shown along with their genomic locations and coding consequences. Nonsynonymous mutation sites among GZ-R6, GZ-R7, GZ-R8 and GZ-HFM01 are indicated in bold.

**Figure 2.**
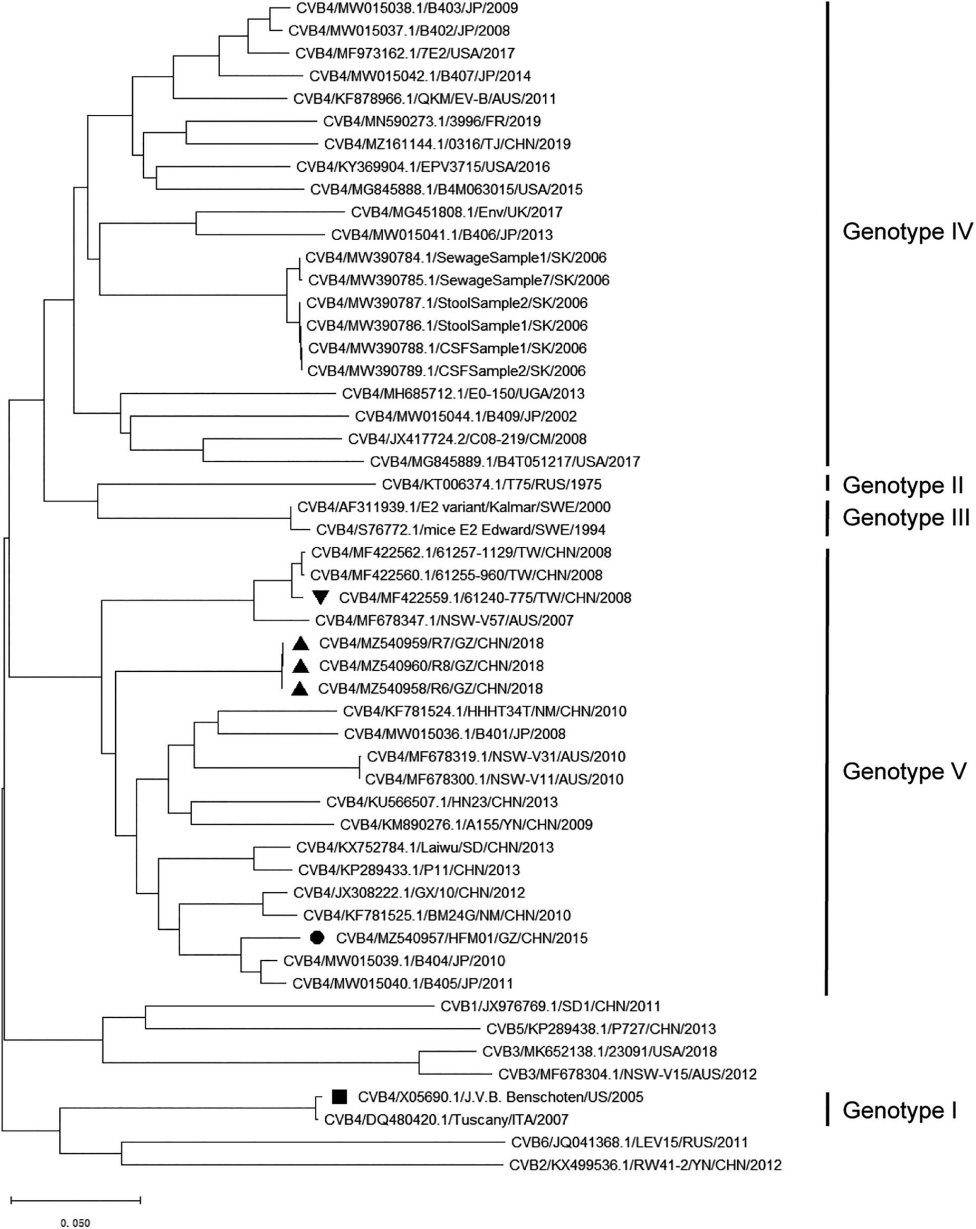
Phylogenetic analysis of genome of coxsackievirus B4 isolates. Three pneumonia-derived coxsackievirus B4 isolates in this research were subjected to genome sequencing analysis to determine their phylogenetic relationships using the neighbour-joining method with 1000 bootstrap replicates implemented in MEGA 11 software. For reference, taxon names include genome type, corresponding GenBank accession number, and country of isolation, strain name, and year of isolation. Strains of coxsackievirus B4 isolated from pneumonia patients in this study are marked with “▴”, the HFMD-derived strain GZ-HFM01 is marked with “●”, a previous reported pneumonia-derived strain with complete ORF is marked with “▾” and the prototype strain is marked with “▪”.

As of May 1, 2023, a total of 46 CVB4 genomes containing complete ORF regions have been uploaded to Genbank. The disease type, country, isolation year, and corresponding reported literature of the isolated strains are shown in Supplementary Table S3. There was only one CVB4 strain, 61255-775, isolated from pneumonia patients with a complete ORF but incomplete UTR (isolated from Taiwan, China, and accession no. MF422559) [18]. The phylogenetic analyses of genomic sequences showed that isolates GZ-R6 to – R8, HFM01, and 61255-775 are all clustered in the same genotype V (Figure 2), consistent with the phylogenetic tree created based on 86 VP1 gene sequences of typical strains of CVB4 genotypes I – V (Supplementary Figure S2).

The reverse genetic systems of GZ-R6 and GZ-HFM01 have been constructed successfully and the virus rescued, demonstrating the integrity of its genome (Supplementary Figure S3). This study is the first to report the full-length genome of CVB4 from patients with a respiratory disease.

### Plaque features and proliferation dynamics of CVB4 isolates in SH-SY5Y cells

**Figure 3.**
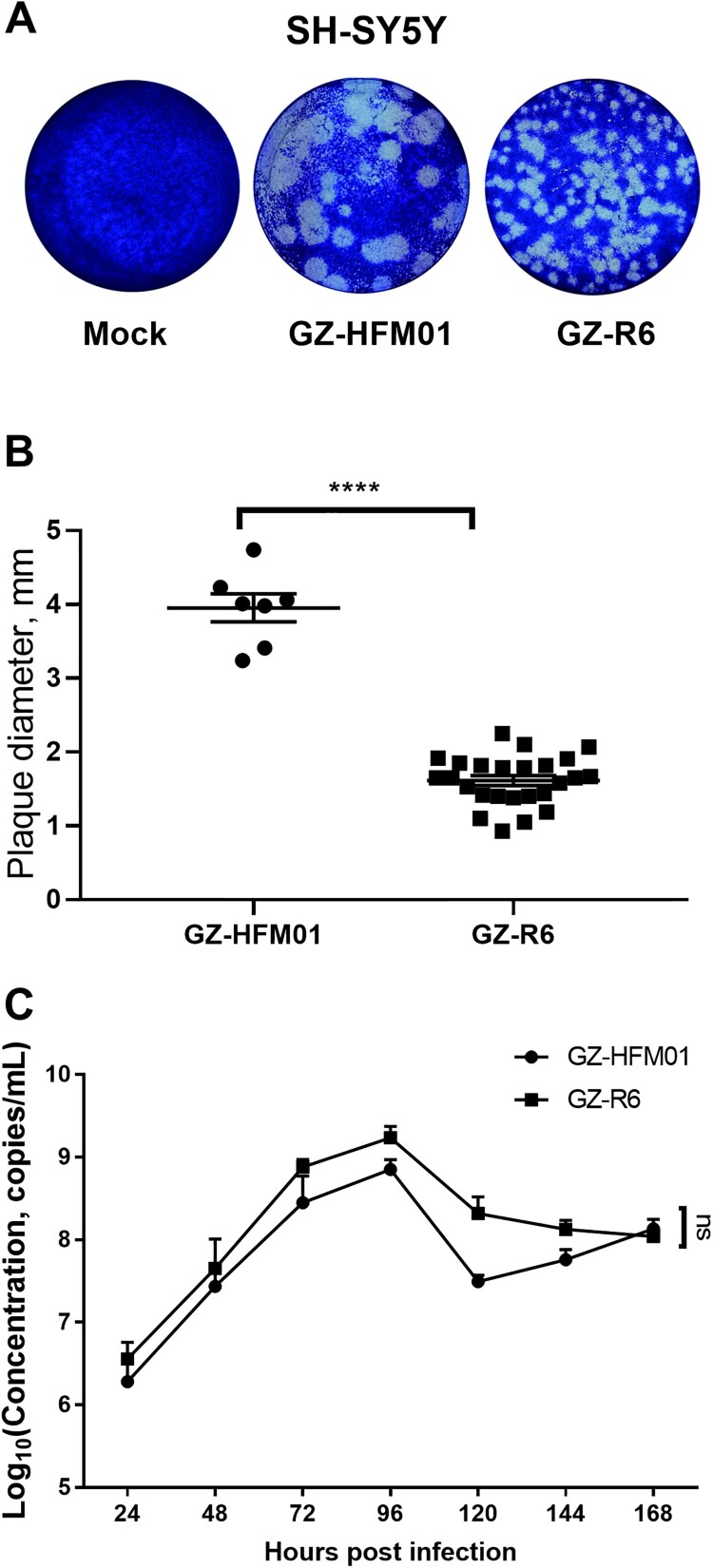
Plaque formation (A), plaque size distribution (B) and the proliferation curve (C) of the pneumonia-derived CVB4 GZ-R6 and the reference HFMD-derived coxsackievirus B4 GZ-HFM01 infection in SH-SY5Y cells. Plaque plates were incubated and stained with crystal violet for 3 days in SH-SY5Y cells in six-well culture plates. Plaque sizes are shown as mean values with SEM and performed by Mann – Whitney U test. Virus genome copies were quantified by qPCR at different hours post-infection (n = 3). Statistical analysis was conducted by ANOVA. ns, not significant; ****, *p* < 0.0001.

CVB4 can break through the blood–brain barrier and infect nerve tissue, leading to severe encephalitis and even death [40]. To analyze the infectivity and pathogenicity of pneumonia-derived CVB4 in neural cells in this study, the representative isolate GZ-R6 was used to infect SH-SY5Y cells, and the characteristics of plaque formation and the proliferation curve were observed and compared with those of the HFMD-derived strain GZ-HFM01. The results showed that the plaques of GZ-HFM01 were significantly larger than those of GZ-R6 (*p* < 0.0001) (Figure 3A, 3B). The proliferation features of the two viruses in SH-SY5Y cells were similar (*p* > 0.05) (Figure 3C). The results indicated that the infectivity and pathogenicity of GZ-HFM01 in neural cells were more potent than those of GZ-R6. There was no significant difference in plaque size between the two viruses in Hela cells (*p* > 0.05) (Supplementary Figure S4), and viral titres were quantified by plaque counting (plaque forming units, pfu) in Hela cells in the following study.

### Infection and pathogenic properties of CVB4 in HAE

**Figure 4.**
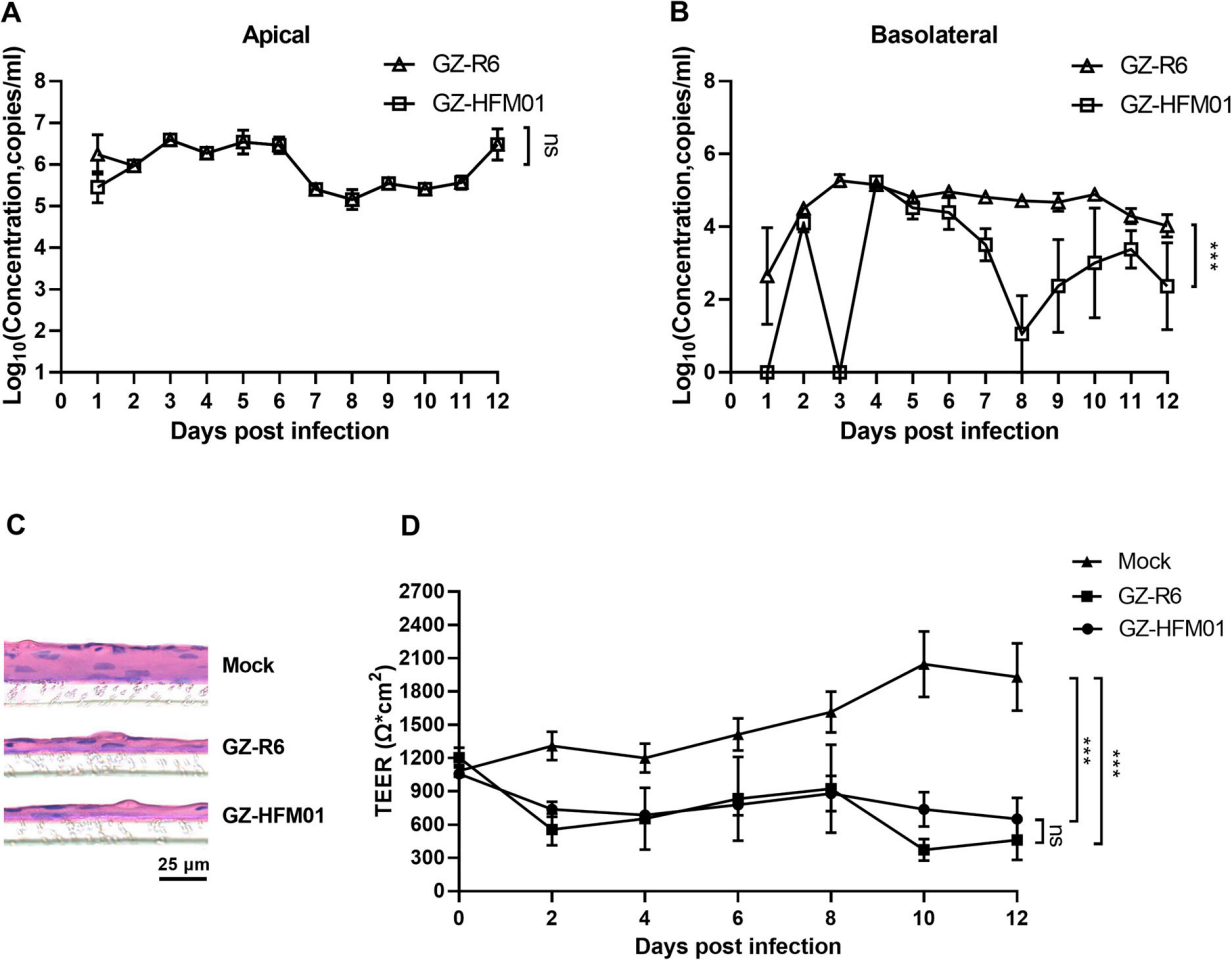
Pneumonia-derived and HFMD-derived coxsackievirus B4 infection in HAE were persistent and caused cytopathogenic effects. GZ-R6 and GZ-HFM01 infected HAE from the apical surface. At the indicated days p.i., the proliferation curves of the two viruses at the apical side (A) and basolateral side (B). At 8 days p.i., coxsackievirus B4-infected HAE membranes taken from the bottom of the inserts were embedded in paraffin, sectioned, and stained using hematoxylin and eosin (C). The transepithelial electrical resistance (TEER) of mock – and coxsackievirus B4-infected HAE was measured at 8 d.p.i. (D). Data are shown as mean values with SEM (n = 4). Statistical analysis was performed by ANOVA test. ns, not significant; ***, *p* < 0.001.

To analyze the infectivity and pathogenic effects of pneumonia-derived and HFMD-derived CVB4, a well-differentiated tissue-like HAE was established and used as a model to mimic the human airway epithelium. After infection via the apical interface, both disease-derived CVB4 strains were released from the apical and basolateral sides of HAE and led to significant cytopathic effects (Figure 4). The proliferation curves of GZ-R6 and GZ-HFM01 from the apical surface of HAE had high similarity and reached the plateau stage very quickly at about 2 d.p.i. (Figure 4A), while more GZ-R6 were released than GZ-HFM01 from the basolateral surface (Figure 4B, *p* < 0.001). From a histological analysis of mock-infected vs. CVB4-infected HAE, we concluded both strains of CVB4 caused tissue damage, and HAE transformed from pseudostratified cells to monolayer cells (Figure 4C). We further monitored the TEER values during infection and found that the values were significantly decreased in HAE with CVB4 infection compared with mock infection (*p* < 0.001). No significant differences were found between the two disease-derived CVB4 (*p* > 0.05) (Figure 4D). However, the TEER values of HAE infected with GZ-R6 were approximately 370 and 190 Ω^.^cm^2^ lower than those infected with GZ-HFM01 at 10 and 12 d.p.i., respectively.

**Figure 5.**
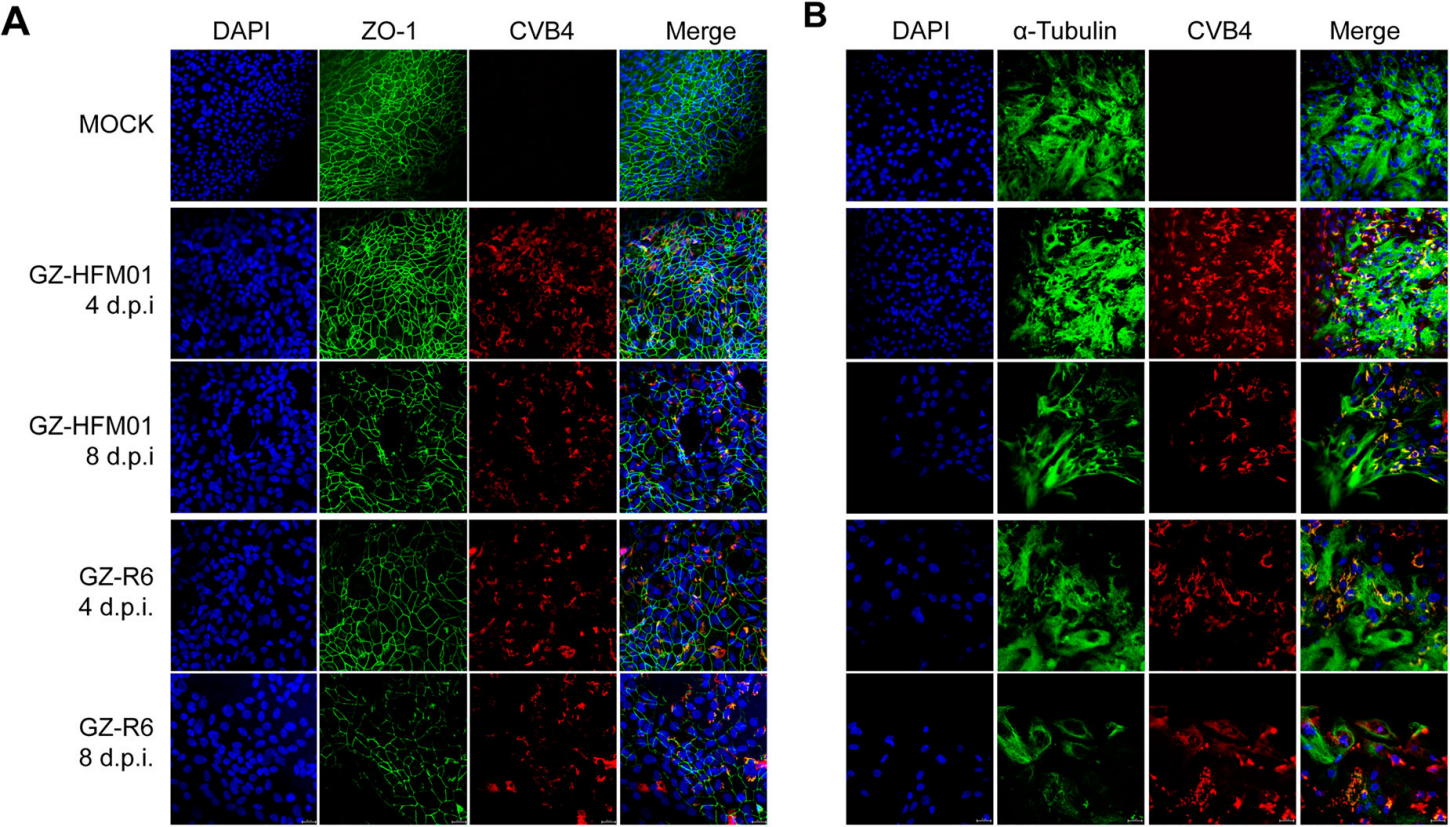
Immunofluorescence analysis of the tight junction protein ZO-1 and the cilia marker α-tubulin during pneumonia-derived coxsackievirus B4 GZ-R6 and HFMD-derived coxsackievirus B4 GZ-HFM01 infection of HAE. Mock – and CVB4-infected HAE cultures at the indicated days post-infection (d.p.i.) were co-stained with anti-CVB4 and anti-ZO-1 antibodies (A), or co-stained with anti-coxsackievirus B4 and anti-α-tubulin antibodies (B). Confocal images were taken at a magnification of ×40. Nuclei were stained with DAPI (blue).

To confirm a role for CVB4 infection in the disruption of the barrier function of the epithelium, ZO-1 was examined at 4 and 8 d.p.i. and compared with the GZ-HFM01 and mock-infection models. Overall, infected HAE showed increased dissociation of ZO-1 from the periphery of the cells compared with mock-infected HAE (Figure 5A), which likely plays a role in reducing TEER. Although there was no significant change in the overall TEER values after the two different disease-derived CVB4 infections (*p* > 0.05), the immunofluorescence results demonstrated the dissociation of ZO-1 in GZ-R6-infected HAE was more intense and rapid than that of GZ-HFM01-infected HAE at 4 and 8 d.p.i. (Figure 5A), which is consistent with the lower TEER values at 10 and 12 dpi in GZ-R6-infected HAE (Figure 4D). Cumulatively, these results demonstrate that GZ-R6 infection disrupts the integrity of HAE, that this may involve the breakdown of polarity and the redistribution of the tight junction protein ZO-1, and the degree of damage was higher than that of GZ-HFM01.

To confirm a role for CVB4 infection in the loss of cilia, we examined the expression of α-tubulin. In GZ-R6-infected HAE, the expression of α-tubulin was drastically decreased at 4 and 8 d.p.i., in contrast to that in mock-infected HAE and similar to the situation with ZO-1, damage to α-tubulin in GZ-R6-infected HAE was more intense and occurred earlier than that in GZ-HFM01-infected HAE (Figure 5B). These results confirmed that CVB4 infection caused the loss of cilia in HAE, and direct tissue damage by GZ-R6 was greater than that of GZ-HFM01.

Notably, GZ-R6-infected HAE showed more significant changes to nuclear enlargement than that of mock – and GZ-HFM01-infected HAE and became obvious at 8 d.p.i. (Figure 5, DAPI), indicating airway epithelial cell hypertrophy.

Collectively, our results showed that CVB4 infection disrupted the tight junction barrier, led to the loss of cilia, and caused airway epithelial cell hypertrophy. These are hallmarks of respiratory tract injury when a loss of epithelial cell polarity occurs, and the pneumonia-derived CVB4 induced more significant damage than the HFMD-derived CVB4.

### Different cytokine distributions of HAE infected with two different disease-derived CVB4 strains

**Figure 6.**
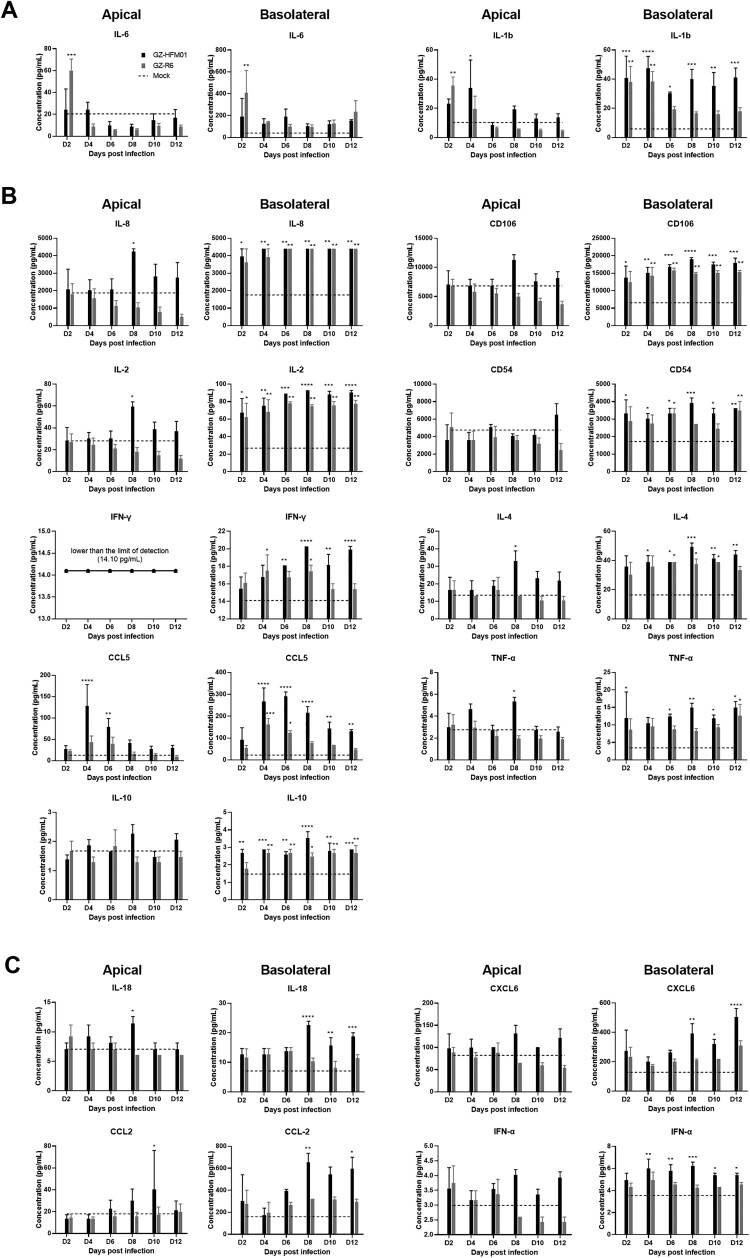
Cytokines secretion of HAE with pneumonia-derived coxsackievirus B4 GZ-R6 and HFMD-derived coxsackievirus B4 GZ-HFM01 infection. IL-6 and IL-1b secretion increased in both apical and basolateral sides rapidly in 2 to 4 d.p.i. in HAE with GZ-R6 infection comparing with mock – and GZ-HFM01 infection, and then decreased immediately (A); cytokines increased significantly in the basolateral but not apical side in GZ-R6 infected HAE, while stronger secretion from both sides occurred in GZ-HFM01-infected HAE, especially in the basolateral direction (B); both sides had no obvious differences in HAE with GZ-R6 infection but increased in GZ-HFM01 infection (C). Statistical analysis compared with mock-infection was performed by ANOVA test. *, *p* < 0.05; ** *p* < 0.01; ***, *p* < 0.001; ****, *p* < 0.0001.

A total of 18 cytokines were detected every two days within 12 days after infection, and samples from both the apical and basolateral sides were tested simultaneously. IL-33, IL-27, and TIMP-1 were lower than the limit of detection or showed no significant change from the mock infection model on both sides (data not shown). The other 15 cytokines showed significant differences (*p* < 0.05) compared to the mock infection (Figure 6). After GZ-R6 infection of HAE, IL-6 and IL-1b significantly increased at both sides during the early infection stage at 2 d.p.i., and then decreased rapidly (Figure 6A); however, IL-8, CD106, IL-2, CD54, IFN-γ, IL-4, CCL5, TNF-α and IL-10 showed significant increases only at the basolateral sides at different infection times, and no differences were observed at the apical side (Figure 6B). The secretion of the remaining four cytokines, IL-18, CXCL6, CCL2 and IFN-α, at both the apical and basolateral sides did not show any significant changes (Figure 6C). Compared to the GZ-R6-infected HAE, GZ-HFM01-infected HAE had a significant increase in all 14 cytokines, except for IL-6, at the basolateral sides. Among them, IL-1b, IL-8, IL-2, IL-4, CCL5, TNF-α and IL-18 increased significantly at both sides of the HAE (the significant increase appeared relatively late on the apical side, mostly at 8 d.p.i.). Overall, the cytokine changes caused by GZ-HFM01 infection were more significant compared to those in the GZ-R6 infection, indicating that GZ-HFM01 infection may cause more severe immune damage to the airways.

## Discussion

CVB4 is associated with one of the highest proportions of reports with fatal outcomes compared with any other EV serotypes, and it can cause a variety of severe diseases [8,11,41]. The high variability, intertypic recombination ability, and high fatality rate of CVB4 demonstrate the importance of continuous monitoring [11,13,14,23]. However, which severe respiratory diseases are caused by CVB4 infection, and their pathogeneses, remain unclear. Our lack of knowledge about CVB4 infections has affected our prevention and treatment capacity. In this study, we monitored patients with severe pneumonia for infection with CVB4 and analyzed the clinical manifestations, viral genome characteristics, and pathogenic properties.

In this study, 7.2% (3/42) of severe pneumonia patients were infected with CVB4, and there were no other common respiratory pathogens, indicating a relatively high prevalence in children [27]. A striking feature of CVB4 infection is that patients infected with different strains often have different clinical manifestations [8,42]. In previous reports, CVB4 was in endemic circulation, genotype V dominated in China, and the main clinical manifestation of infected patients was HFMD [13,14,40]. Three pneumonia-derived CVB4 isolates obtained in this study and the reference HFMD-derived strain GZ-HFM01 all clustered to genotype V according to the full-length genomes and VP1 phylogenetic trees. But the clinical manifestations of the positive patients differed significantly. The virulence of a virus is closely related to the proteins it encodes. The genome sequences of the three CVB4 isolates in this study had high similarity but differed significantly from GZ-HFM01, which is an important factor leading to differences in their clinical manifestations.

An HAE model was used to further investigate the pathogenic characteristics of CVB4 in human airways and to compare differences between pneumonia-derived CVB4 and HFMD-derived CVB4. The respiratory epithelium comprises polarized cells at the interface between the environment and airway tissues. The polarized ciliated primary HAE, which is generated by growing isolated human bronchial/tracheal epithelial cells at an ALI for an average of one month, forms a pseudo-stratified, mucociliary epithelium and displays morphologic and phenotypic characteristics resembling those of the *in vivo* human airway epithelium of the lungs [43–45]. Recent studies have revealed that this model system recapitulates important characteristics of interactions between respiratory viruses and their host cells [35,46–49]. CVB4 infection disrupted the tight junction barrier, leading to the loss of cilia and airway epithelial cell hypertrophy. Compared with HFMD-derived CVB4, the direct tissue damage caused by pneumonia-derived CVB4 is more intense and rapid, and the acute damage to HAE polarity is an important reason for the more prominent manifestation of respiratory diseases.

On the other hand, HAE infected with CVB4 released the virus from both the apical and basolateral sides, and significantly more pneumonia-derived CVB4 than HFMD-derived CVB4 was released from the basolateral side, which might also be related to the more severe barrier damage caused by pneumonia-derived CVB4. The respiratory epithelium basolateral side juxtaposes deeper lung tissues. The basolaterally secreted virus impacts the lung’s sub-epithelial structures and cells, such as serous/mucous glands, fibroblasts, blood vessels, and immune cells [50]. The strong ability of pneumonia-derived CVB4 to release progeny from the basolateral side may make it easier for the virus to enter the bloodstream, increasing the risk of multiple organ damage, which is consistent with the detection of liver injury-related indexes in all three CVB4 positive patients in this study.

In addition to the direct tissue damage caused by virus replication, immune damage is an important pathogenic mechanism in viral infection. Although the HAE model lacks immune synergistic ability, it can be partially studied through changes to the secretion of related cytokines after infection. This study analyzed changes in the secretion of 18 cytokines in bilateral HAE infected with CVB4. Overall, pneumonia-derived CVB4 caused significant increases in two important pro-inflammatory factors, IL-6 and IL-1b, on both sides in the early stages of infection. This is consistent with the high levels of IL-6 detected in three patient's plasma, which may lead to a rapid immune response in the body in the short term. Except for these two cytokines, pneumonia-derived CVB4 did not cause a significant increase in cytokines on the apical side of HAE. In contrast, HFMD-derived CVB4 caused significant increases in the secretion of multiple cytokines on both sides of HAE, and the secretion of cytokines on the basolateral side was also generally stronger than that of the pneumonia-derived CVB4 model. From the overall distribution of cytokines, it is speculated that the sustained immune damage caused by HFMD-derived CVB4 may be stronger than that caused by pneumonia-derived CVB4, but this requires more research, especially animal experiments, to verify.

CVB4 are cytolytic viruses, but they are also able to establish a persistent infection in human pancreatic islets [8]. The virus can enter the bloodstream and impact multiple organs and can break through the blood–brain barrier and infect nerve tissue, leading to severe encephalitis and even death [40]. Previous studies have shown that severe nerve damage is the main factor leading to death from CVB4 infection [11,40,41]. To further analyze whether there is a difference in neurovirulence between pneumonia-derived CVB4 and HFMD-derived CVB4, SH-SY5Y cells commonly used for studying EV-induced nerve injury *in vitro* [51] were infected with CVB4. The plaques of HFMD-derived CVB4 in SH-SY5Y cells were significantly larger than those of pneumonia-derived CVB4, but their proliferation curves were similar. This indicated that the neurovirulence of HFMD-derived CVB4 is higher than that of pneumonia-derived CVB4, which is consistent with the neurological manifestations in patients infected with these two viruses.

There is a lack of research on the virulent amino acids of CVB4. Previous reports proved that Thr-129 VP1 and Arg-16 VP4 are important virulent determinants associated with the development of hypoglycemia and hyperamylasemia [38,39]. However, the GZ-R6 to – R8 and GZ-HFM01 in this study did not have these two determinants, which is consistent with the clinical absence of pancreatic-related diseases. The virulent determinants of EV not only existed in the coding region but also in the UTR [52]. In this study, the full-length genomes of pneumonia-derived CVB4 were obtained for the first time, providing an important basis for further in-depth studies into virulent determinants.

Additionally, it is worth noting that platelets are elevated in patients with severe CVB4 pneumonia infections. There are reports that CVB4 can cause intracardiac thrombosis and lead to death [53], and the elevation of platelets may provide conditions for thrombosis. In addition, the 2A protease of CVB1 can cleave the thrombin recognition LVPRGS motif [54], and further research is needed to determine whether CVB4 has the same ability.

Overall, pneumonia-derived CVB4 infection can cause severe human airway epithelial damage and polarity disruption, and the inflammatory cytokines IL-6 and IL-1b significantly increase in the early stages of infection, suggesting that early antiviral, inflammatory control interventions may be important for disease treatment, but more in-depth studies are needed.

This study investigated CVB4 infections in patients with severe pneumonia and their pathogenic characteristics, enriching our knowledge of CVB4 infection and providing necessary information for its prevention and treatment. However, there were also some limitations. The study only monitored CVB4 infections in patients with severe pneumonia in the short term, and there are still a lack of monitoring data on the infection status of outpatient and healthy populations, making it impossible to obtain a comprehensive profile of the prevalence of CVB4. Research on the pathogenic characteristics of infection is mainly conducted using HAE, and some results still need to be further validated in animals.

## Supplementary Material

Supplemental Material

Supplemental Material

Supplemental Material

Supplemental Material

Supplemental Material

## Data Availability

All data generated or analyzed during this study are included in this published article and its supplementary information files. The full-length genomes of CVB4 isolates identified in this study were deposited in GenBank under the accession numbers MZ540957 – MZ540960.
