# Supplementary material for: Severe pneumonia and pathogenic damage in human airway epithelium caused by Coxsackievirus B4
Source: Emerg Microbes Infect. 2023 Sep 27;12(2):2261560. doi: 10.1080/22221751.2023.2261560 (PMC10538465; doi:10.1080/22221751.2023.2261560)
Supplement: Supplemental Material [file TEMI_A_2261560_SM6339.docx]

**Supplementary materials**

**Figure legends**

**Figure S1. The cytopathic phenotype of pneumonia-derived coxsackievirus B4 strain GZ-R6 and HFMD-derived coxsackievirus B4 strain GZ-HFM01 in Hela cells**

**Figure S2. Phylogenetic analysis of VP1 gene of coxsackievirus B4 isolates**

VP1 genes of three coxsackievirus B4 isolates were subjected to nucleotide sequencing analyses using the neighbour-joining method with 1000 bootstrap replicates implemented in MEGA 11 software. For reference, taxon names include genome type, corresponding GenBank accession number, and country of isolation, strain name, and year of isolation. Strains of coxsackievirus B4 isolated from patients in this study are marked with “●”.

**Figure S3. Schematic of construction of coxsackievirus B4 isolates GZ-R6 and GZ-HFM01 reverse genetic system**

Genome map of the constructed system (A, not to scale). Full-length cDNA obtained from GZ-R6 and GZ-HFM01 using RT-PCR was constructed in pcDNA3.1(+) with 25nt polyA between hammerhead-type ribozyme (HAMRz) and Hepatitis delta virus ribozyme (HDVRz); The viruses were rescued by transfection of the infective plasmid into Hela cells, and the supernatant from cell freeze-thaw lysis led to cytopathic effects in naïve Hela cells (B and C). The viruses were recognized by coxsackievirus B4-specific antibody (D).

**Figure S4. Plaque formation (A) and size distribution (B) of the representative coxsackievirus B4 isolate GZ-R6 and the reference GZ-HFM01 infection in Hela cells**

Plaque plates were incubated and stained with crystal violet for 2 days in Hela cells in six-well plates. Data are shown as mean values with SEM. Statistical analysis was performed by Mann–Whitney U test. ns, not significant.

**Table S1. Primer and probe for enterovirus and types screening**

| **Target** | **Primer and probe** | **Sequence (5**′**–3**′**)**^a^ |
| --- | --- | --- |
| **qPCR testing for pan-EV and types** | | |
| Pan-EV | EVF | GAAGAGTCTATTGAGCTARTTRGTAGTCCT |
|  | EVR | GAAACACGGACACCCAAAGTAGT |
|  | EVProbe | FAM-CGGCCCCTGAATGCGGCTAATC-BHQ1 |
| CVB4 | CVB4F | CCTGTTAAAGACGTGATG |
|  | CVB4R | GTTGCCAAGTGTTATTGA |
|  | CVB4Probe | FAM-CGCTTAACTCTCCAACAGTGG-BHQ1 |
| EV71 | EV71F | CACAAGCCAGCGGGTAGTG |
|  | EV71R | AAACACGGACACCCAAAGTAGTC |
|  | EV71FB | FAM- TCGTAACGGGTAACTCTGCAGCGGA-BHQ1 |
| CVA16 | CVA16F2 | TCCTTTCGACTCGGCTCTCA |
|  | CVA16R2 | TTCAGATGTAGCGCCTGTATTGA |
|  | CVA16FB2 | FAM-TTTGGTCTACTGGTCATCCCGGTGGT-BHQ1 |
|  |  |  |
| **Typing primes for 450 VP1 fragment** | | |
| Typing primers | TypeF | GCICCIGAYTGITGICCRAA |
|  | TypeR1 | ATGTAYRTICCIMCIGGIGC |
|  | TypeR2 | ATGTAYGTICCICCIGGIGG |

^a^ R represents A or G; Y represents C or T; M represents A or C. I represent Hypoxanthine.

**Table S2. Comparative 5′ and 3′ untranslated region (UTR) analysis of coxsackievirus B4 isolates of GZ-R6, GZ-R7, GZ-R8 and GZ-HFM01 with prototype J.V.B. Benschoten**

| **Genome region** | **nt** | **J.V.B. Benschoten** | **GZ-HFM01** | **GZ-R6** | **GZ-R7** | **GZ-R8** |
| --- | --- | --- | --- | --- | --- | --- |
| **5'-UTR** | 22 | A | T | T | T | T |
|  | 40 | A | C | C | C | C |
|  | 62 | T | C | C | C | C |
|  | 63 | C | A | A | A | A |
|  | 64 | C | A | A | A | A |
|  | 71 | T | T | C | C | C |
|  | 89 | A | T | T | T | T |
|  | 90 | A | T | T | T | T |
|  | 94 | C | T | T | T | T |
|  | 95 | C | T | T | T | T |
|  | 102 | T | A | A | A | A |
|  | 104 | C | - | - | - | - |
|  | 106 | C | T | T | T | T |
|  | 119 | A | G | G | G | G |
|  | 120 | A | C | C | C | C |
|  | 121 | G | A | G | G | G |
|  | 122 | A | C | C | C | C |
|  | 123 | A | A | T | T | T |
|  | 124 | A | C | C | C | C |
|  | 128 | T | - | - | - | - |
|  | 130 | G | A | A | A | A |
|  | 131 | T | C | C | C | C |
|  | 135 | T | C | C | C | C |
|  | 136 | T | A | A | A | A |
|  | 137 | A | G | G | G | G |
|  | 139 | T | A | A | A | A |
|  | 141 | A | G | G | G | G |
|  | 143 | G | A | G | G | G |
|  | 144 | C | T | T | T | T |
|  | 145 | A | G | G | G | G |
|  | 149 | A | C | C | C | C |
|  | 150 | C | A | A | A | A |
|  | 155 | C | C | T | T | T |
|  | 156 | T | C | C | C | C |
|  | 157 | G | A | A | A | A |
|  | 161 | T | C | C | C | C |
|  | 162 | T | G | A | A | A |
|  | 166 | C | T | T | T | T |
|  | 171 | T | C | C | C | C |
|  | 180 | G | A | A | A | A |
|  | 181 | T | A | A | A | A |
|  | 203 | G | A | A | A | A |
|  | 209 | T | C | C | C | C |
|  | 210 | G | A | A | A | A |
|  | 216 | C | T | T | T | T |
|  | 217 | T | C | T | T | T |
|  | 228 | C | G | A | A | A |
|  | 232 | T | C | C | C | C |
|  | 248 | T | T | C | C | C |
|  | 259 | G | A | A | A | A |
|  | 277 | C | A | A | A | A |
|  | 278 | G | A | A | A | A |
|  | 282 | A | C | C | C | C |
|  | 284 | G | A | G | G | G |
|  | 285 | A | G | A | A | A |
|  | 302 | T | A | A | A | A |
|  | 308 | C | A | A | A | A |
|  | 315 | T | A | A | A | A |
|  | 336 | G | A | T | T | T |
|  | 345 | G | A | A | A | A |
|  | 349 | G | A | A | A | A |
|  | 352 | C | T | T | T | T |
|  | 381 | T | C | C | C | C |
|  | 382 | G | A | A | A | A |
|  | 388 | C | T | T | T | T |
|  | 389 | A | G | A | A | A |
|  | 394 | G | A | A | A | A |
|  | 395 | C | T | T | T | T |
|  | 396 | A | G | G | G | G |
|  | 404 | C | T | T | T | T |
|  | 405 | T | C | C | C | C |
|  | 406 | G | A | A | A | A |
|  | 411 | A | G | G | G | G |
|  | 428 | C | T | T | T | T |
|  | 447 | G | T | T | T | T |
|  | 482 | C | T | T | T | T |
|  | 489 | C | T | T | T | T |
|  | 490 | A | G | G | G | G |
|  | 493 | T | C | C | C | C |
|  | 493 | T | C | C | C | C |
|  | 494 | G | C | C | C | C |
|  | 496 | A | C | C | C | C |
|  | 499 | G | T | T | T | T |
|  | 505 | C | A | A | A | A |
|  | 507 | A | G | G | G | G |
|  | 510 | G | A | A | A | A |
|  | 512 | T | C | C | C | C |
|  | 513 | G | A | G | G | G |
|  | 526 | C | T | T | T | T |
|  | 532 | T | C | C | C | C |
|  | 546 | G | C | C | C | C |
|  | 573 | A | A | G | G | G |
|  | 578 | T | C | C | C | C |
|  | 579 | A | G | A | A | A |
|  | 580 | C | T | T | T | T |
|  | 581 | C | A | A | A | A |
|  | 609 | A | G | G | G | G |
|  | 639 | T | C | C | C | C |
|  | 642 | A | G | G | G | G |
|  | 646 | T | A | A | A | A |
|  | 657 | A | G | G | G | G |
|  | 660 | C | A | A | A | A |
|  | 663 | A | C | C | C | C |
|  | 668 | T | A | A | A | A |
|  | 669 | G | A | A | A | A |
|  | 672 | T | T | C | C | C |
|  | 675 | T | G | G | G | G |
|  | 678 | T | A | A | A | A |
|  | 681 | C | T | T | T | T |
|  | 683 | T | T | A | A | A |
|  | 687 | C | A | A | A | A |
|  | 688 | T | A | A | A | A |
|  | 690 | G | A | A | A | A |
|  | 691 | G | A | A | A | A |
|  | 692 | A | G | G | G | G |
|  | 693 | C | T | C | C | C |
|  | 695 | A | T | T | T | T |
|  | 696 | C | G | G | G | G |
|  | 698 | G | - | - | - | - |
|  | 701 | A | G | G | G | G |
|  | 706 | - | G | G | G | G |
|  | 709 | A | G | G | G | G |
|  | 712 | T | A | A | A | A |
|  | 716 | T | G | A | A | A |
|  | 718 | T | C | C | C | C |
|  | 719 | T | C | T | T | T |
|  | 721 | C | A | A | A | A |
|  | 727 | G | A | A | A | A |
|  | 728 | A | C | C | C | C |
|  | 729 | G | T | C | C | C |
|  | 733 | C | T | C | C | C |
|  | 738 | C | G | G | G | G |
|  | 739 | G | T | T | T | T |
|  | 740 | A | G | G | G | G |
|  | 741 | T | G | T | T | T |
| **3'UTR** | 7295 | A | G | A | A | A |
|  | 7297 | - | A | G | G | G |
|  | 7305 | A | C | C | C | C |
|  | 7306 | - | G | G | G | G |
|  | 7315 | A | T | T | T | T |
|  | 7316 | A | T | T | T | T |
|  | 7317 | C | T | C | C | C |
|  | 7322 | T | C | C | C | C |
|  | 7325 | A | - | - | - | - |
|  | 7341 | T | C | C | C | C |
|  | 7347 | A | C | C | C | C |
|  | 7367 | C | T | T | T | T |
|  | 7368 | A | G | G | G | G |
|  | 7387 | C | C | C | C | A |
|  | 7388 | G | A | A | A | G |
|  | 7389 | T | T | T | T | A |

Differences in nucleic acid are shown along with their genomic locations. -, deletion.

**Table S3. The genome sequences of coxsackievirus B4 with complete ORF submitted in Genbank (as of May 1, 2023)**

| **Accession no.** | **Strain** | **Country** | **Year** | **Isolation source** | **Isolation cells** | **Reference** |
| --- | --- | --- | --- | --- | --- | --- |
| MZ540958 | GZ-R6 | China | 2018 | Throat swab samples from patients with severe pneumonia | Hela cells | This study |
| MZ540959 | GZ-R7 | China | 2018 | Throat swab samples from patients with severe pneumonia | Hela cells | This study |
| MZ540960 | GZ-R8 | China | 2018 | Throat swab samples from patients with severe pneumonia | Hela cells | This study |
| MZ540957 | GZ-HFM01 | China | 2015 | Stool samples from hand-foot and mouth disease | LLC-MK2 cells | This study |
| X05690.1 | J.V.B. Benschoten | USA | 1951 | Stool from a child with chest and abdominal pain | / | [1, 2] |
| MF678347.1 | NSW-V57 | Australia | 2007 | Stool | / | [3] |
| MF678319.1 | NSW-V31 | Australia | 2010 | Stool | / | [3] |
| MF678300.1 | NSW-V11 | Australia | 2010 | Stool | / | [3] |
| MN590273.1 | FR/3996 | France | 2019 | Human blood from self-limited meningitis | / | [4] |
| MG451808.1 | Env | UK | 2017 | Sewage | / | [5] |
| KX752784.1 | Laiwu/SD | China | 2013 | Aseptic meningitis | / | [6] |
| KU566507.1 | HN23 | China | 2013 | HFMD | RD cells | / |
| KP289433.1 | P11 | China | 2013 | Cerebrospinal fluid from hand,foot,and mouth disease | / | [7] |
| AF311939.1 | E2 variant | Sweden | 2000 | pancreatic tissue from ketoacidosis | / | [8] |
| KF878966.1 | QKM/EV-B | Australia | 2011 | Homo sapiens | / | / |
| KT006374.1 | T75 | Russia | 1975 | Sus scrofa domesticus (swine) | IBRS cells | [9] |
| MF973162.1 | 7E2 | USA | 2017 | Homo sapiens | / | / |
| JX308222.1 | GX/10 | China | 2010 | throat swabs from a child with hand, foot, and mouth disease complicated with aseptic meningitis and myocarditis | / | [10] |
| KF781525.1 | BM24G/NM | China | 2010 | throat swabs from hand, foot, and mouth disease | Hep-2 cells | [11] |
| KF781524.1 | HHHT34T | China | 2010 | throat swabs from hand, foot, and mouth disease | Hep-2 cells | [11] |
| DQ480420.1 | Tuscany | Italy | 2006 | Pancreatic islets | / | [12] |
| MW390784 | Sewage Sample1 | Slovakia | 2006 | Sewage Sample1 | RD cells | [13] |
| MW390785 | Sewage Sample7 | Slovakia | 2006 | Sewage Sample7 | RD cells | [13] |
| MW390786 | stool sample 1 | Slovakia | 2006 | stool sample 1 from Homo sapiens | RD cells | [13] |
| MW390787.1 | stool sample 2 | Slovakia | 2006 | stool sample 2 from Homo sapiens | RD cells | [13] |
| MW390788.1 | CSFSample1 | Slovakia | 2006 | Cerebrospinal fluid(CSFSample1) | RD cells | [13] |
| MW390789.1 | CSFSample2 | Slovakia | 2006 | Cerebrospinal fluid(CSFSample2) | RD cells | [13] |
| KY369904.1 | EPV3715 | USA | 2016 | Homo sapiens | / | / |
| MZ161144.1 | 0316/TJ | China | 2019 | Stool specimen from HFMD | RD cells | [14] |
| JX417724.2 | C08-219 | Cameroon | 2008 | Feces from Homo sapiens | / | [15] |
| S76772 | E2 Edward | Sweden | 1994 | Type 1 diabetes | mice | [16] |
| MW015041 | B406 | Japan | 2013 | Raw sewage | Vero cells | [17] |
| MW015044 | B409 | Japan | 2002 | River | Hep-2 cells | [17] |
| MH685712 | E0-150 | Uganda | 2013 | Nasopharynx from pediatric febrile illness | / | [18] |
| MG845889 | B4T051217 | USA | 2017 | Raw sewage | / | [19] |
| MG845888 | B4M063015 | USA | 2015 | Raw sewage | / | [19] |
| MW015042 | B407 | Japan | 2014 | raw sewage | Vero cells | [17] |
| MW015038 | B403 | Japan | 2009 | raw sewage | MA104 cells | [17] |
| MW015037 | B402 | Japan | 2008 | raw sewage | Vero cells | [17] |
| MW015036 | B401 | Japan | 2008 | raw sewage | MA104 cells | [17] |
| MW015040 | B405 | Japan | 2011 | raw sewage | Vero cells | [17] |
| MW015039 | B404 | Japan | 2010 | raw sewage | Vero cells | [17] |
| MF422559 | 61240-775 | China | 2008 | pneumonia | / | [20] |
| MF422562 | 61257-1129 | China | 2008 | Acute tonsillitis/fever | / | [20] |
| MF422560 | 61255-960 | China | 2008 | Acute pharyngitis | / | [20] |
| KM890276 | A155 | China | 2009 | Stool sample of a child with aseptic meningitis | A549 cells | [21] |

“/” means direct submission, and no further information was obtained from Genbank or available reports.

**References of Table S3**

1. Jenkins, O., et al., *The complete nucleotide sequence of coxsackievirus B4 and its comparison to other members of the Picornaviridae.* J Gen Virol, 1987. **68 ( Pt 7)**: p. 1835-48.

2. Xiao, J., et al., *Coxsackievirus B4: an underestimated pathogen associated with a hand, foot, and mouth disease outbreak.* Arch Virol, 2021. **166**(8): p. 2225-2234.

3. Isaacs, S.R., et al., *Amplification and next generation sequencing of near full-length human enteroviruses for identification and characterisation from clinical samples.* Sci Rep, 2018. **8**(1): p. 11889.

4. Luciani, L., et al., *Fatal underhanded chronic enterovirus infection associated with anti-CD20 monotherapy for central nervous system demyelinating disease.* Mult Scler, 2021. **27**(2): p. 320-323.

5. Majumdar, M., et al., *Isolation of Vaccine-Like Poliovirus Strains in Sewage Samples From the United Kingdom.* J Infect Dis, 2018. **217**(8): p. 1222-1230.

6. Xu, L., et al., *Genomic characteristics of coxsackievirus B4 isolated in Henan province of China.* Chinese Journal of Viral Diseases, 2018. **8**(1): p. 33-38.

7. Guo, W.P., et al., *Fourteen types of co-circulating recombinant enterovirus were associated with hand, foot, and mouth disease in children from Wenzhou, China.* J Clin Virol, 2015. **70**: p. 29-38.

8. Zhou, M. and F. Li, *Complete nucleotide sequence of a coxsackievirus B4 strain that establishes infection in ICR mice pancreas and induces glucose intolerance.* Anat Rec (Hoboken), 2008. **291**(5): p. 601-9.

9. Lomakina, N.F., et al., *Epizootic of vesicular disease in pigs caused by coxsackievirus B4 in the Soviet Union in 1975.* J Gen Virol, 2016. **97**(1): p. 49-52.

10. Hu, Y.F., et al., *Complete genome sequence of a recombinant coxsackievirus B4 from a patient with a fatal case of hand, foot, and mouth disease in Guangxi, China.* J Virol, 2012. **86**(19): p. 10901-2.

11. Tian, X., et al., *New coxsackievirus B4 genotype circulating in Inner Mongolia Autonomous Region, China.* PLoS One, 2014. **9**(3): p. e90379.

12. Dotta, F., et al., *Coxsackie B4 virus infection of beta cells and natural killer cell insulitis in recent-onset type 1 diabetic patients.* Proc Natl Acad Sci U S A, 2007. **104**(12): p. 5115-20.

13. Benkoova, B., et al., *Coxsackievirus B4 sewage-isolate induces pancreatitis after oral infection of mice.* FEMS Microbiol Lett, 2021. **368**(15).

14. Tan, Z.L., et al., *An imported Genotype D Coxsackievirus B4 Strain Identified in Tianjin, China.* Biomed Environ Sci, 2022. **35**(3): p. 239-247.

15. Sadeuh-Mba, S.A., et al., *High frequency and diversity of species C enteroviruses in Cameroon and neighboring countries.* J Clin Microbiol, 2013. **51**(3): p. 759-70.

16. Kang, Y., et al., *Complete nucleotide sequence of a strain of coxsackie B4 virus of human origin that induces diabetes in mice and its comparison with nondiabetogenic coxsackie B4 JBV strain.* J Med Virol, 1994. **44**(4): p. 353-61.

17. Torii, S., et al., *Genotype-dependent kinetics of enterovirus inactivation by free chlorine and ultraviolet (UV) irradiation.* Water Res, 2022. **220**: p. 118712.

18. Ramesh, A., et al., *Metagenomic next-generation sequencing of samples from pediatric febrile illness in Tororo, Uganda.* PLoS One, 2019. **14**(6): p. e0218318.

19. Meister, S., et al., *Variability in Disinfection Resistance between Currently Circulating Enterovirus B Serotypes and Strains.* Environ Sci Technol, 2018. **52**(6): p. 3696-3705.

20. Chien, Y.S., et al., *Genomic analysis of serologically untypable human enteroviruses in Taiwan.* J Biomed Sci, 2019. **26**(1): p. 49.

21. Zhu, Y., et al., *A Coxsackie B4 virus isolated in Yunnan in 2009 is a recombinant.* Virus Genes, 2015. **50**(3): p. 375-80.
