# Supplementary figures and images for: Severe pneumonia and pathogenic damage in human airway epithelium caused by Coxsackievirus B4
Source: Emerg Microbes Infect. 2023 Sep 27;12(2):2261560. doi: 10.1080/22221751.2023.2261560 (PMC10538465; doi:10.1080/22221751.2023.2261560)

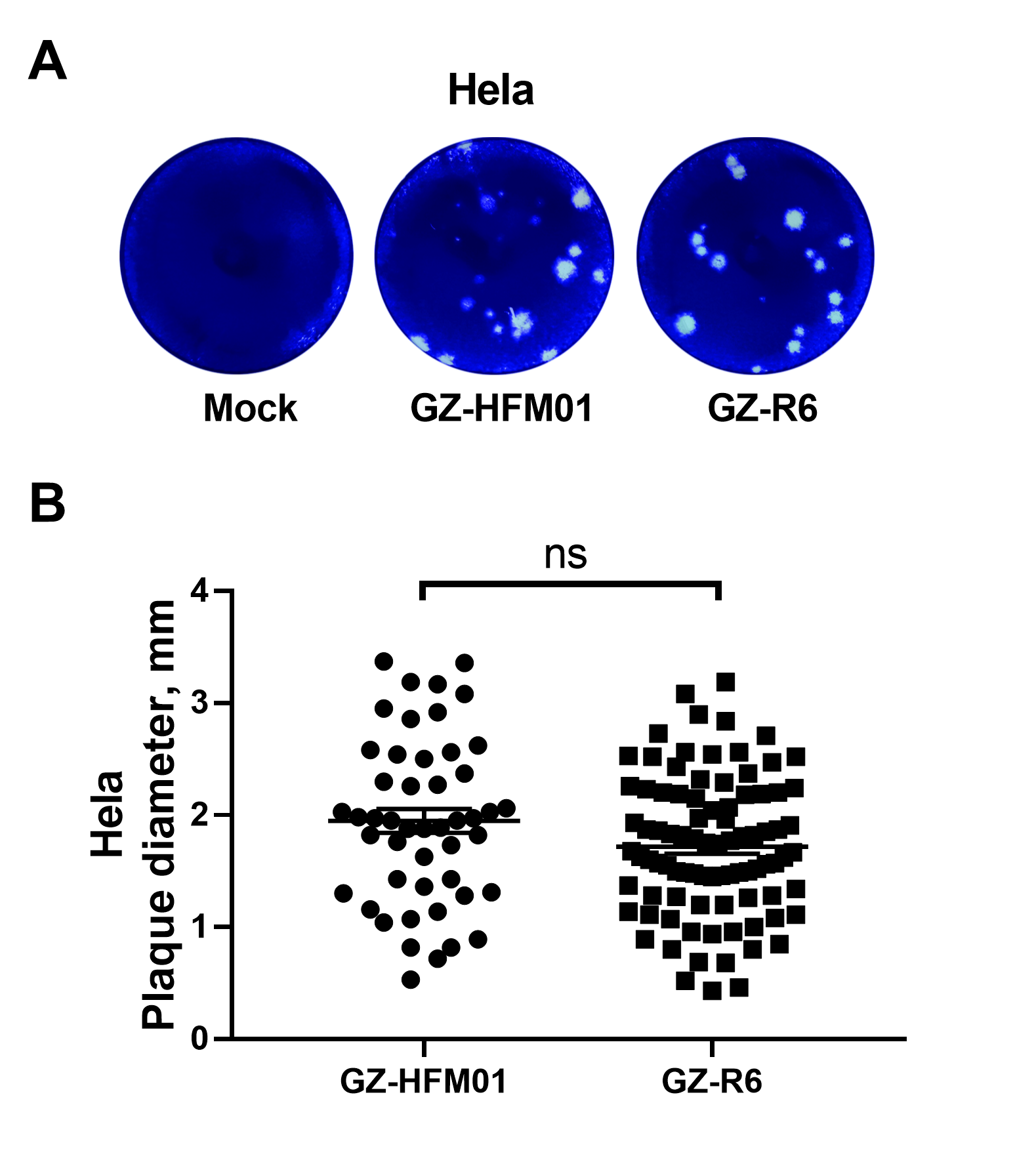

Supplement: Supplemental Material [file TEMI_A_2261560_SM6336.tif]

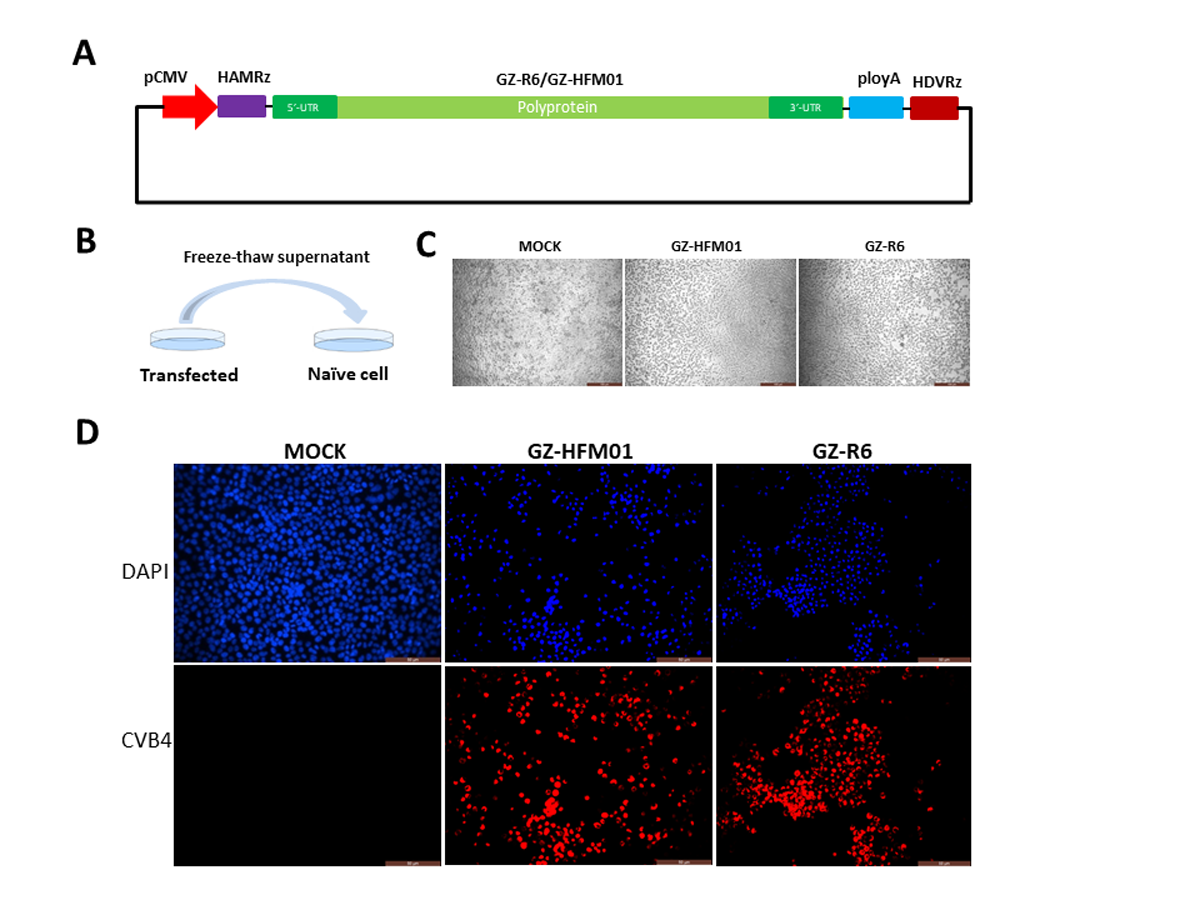

Supplement: Supplemental Material [file TEMI_A_2261560_SM6333.tif]

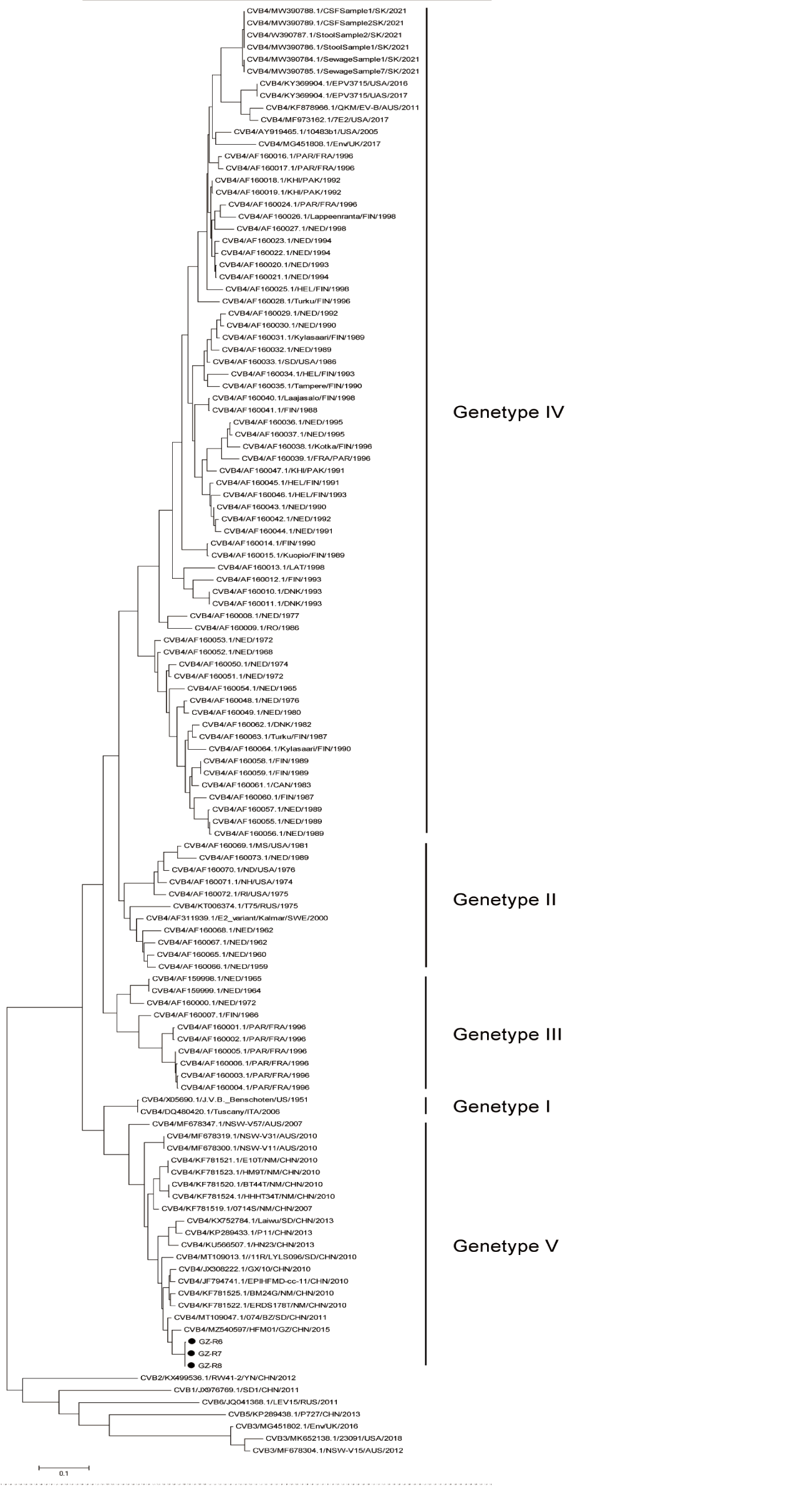

Supplement: Supplemental Material [file TEMI_A_2261560_SM6300.tif]

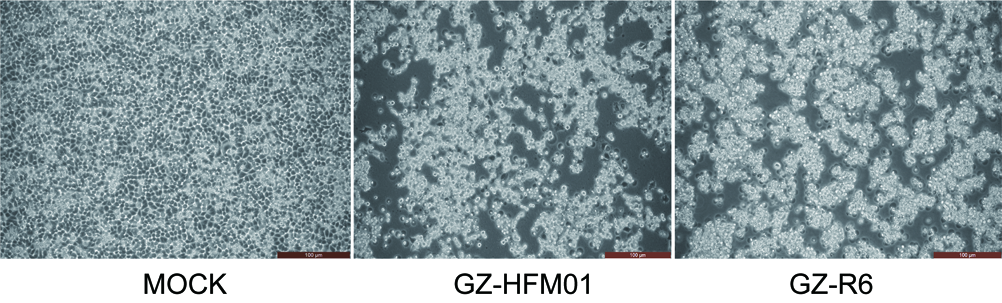

Supplement: Supplemental Material [file TEMI_A_2261560_SM6291.tif]
